# FOX Optimization Algorithm Based on Adaptive Spiral Flight and Multi-Strategy Fusion

**DOI:** 10.3390/biomimetics9090524

**Published:** 2024-08-30

**Authors:** Zheng Zhang, Xiangkun Wang, Li Cao

**Affiliations:** 1 School of Information Engineering, Wenzhou Business College, Wenzhou 325035, China; 2 School of Intelligent Manufacturing and Electronic Engineering, Wenzhou University of Technology, Wenzhou 325035, China

**Keywords:** fox optimization algorithm, variable spiral search strategy, levy flight, tent chaotic mapping, adaptive weights

## Abstract

Adaptive spiral flight and multi-strategy fusion are the foundations of a new FOX optimization algorithm that aims to address the drawbacks of the original method, including weak starting individual ergodicity, low diversity, and an easy way to slip into local optimum. In order to enhance the population, inertial weight is added along with Levy flight and variable spiral strategy once the population is initialized using a tent chaotic map. To begin the process of implementing the method, the fox population position is initialized using the created Tent chaotic map in order to provide more ergodic and varied individual beginning locations. To improve the quality of the solution, the inertial weight is added in the second place. The fox random walk mode is then updated using a variable spiral position updating approach. Subsequently, the algorithm’s global and local searches are balanced, and the Levy flying method and greedy approach are incorporated to update the fox location. The enhanced FOX optimization technique is then thoroughly contrasted with various swarm intelligence algorithms using engineering application optimization issues and the CEC2017 benchmark test functions. According to the simulation findings, there have been notable advancements in the convergence speed, accuracy, and stability, as well as the jumping out of the local optimum, of the upgraded FOX optimization algorithm.

## 1. Introduction

Selecting the best option among a range of alternative solutions is known as an optimization problem, and any issue with many workable solutions falls under this category [1,2]. Numerous domains, including image processing, sensor networks, engineering design, system control, etc., face optimization challenges [3]. Metaheuristic algorithms are often created by drawing inspiration from natural phenomena or analogies, such as modeling the behavior of groups or the development of creatures [4,5]. Its wide applicability is not reliant on the particular mathematical aspects of the issue, having the ability to go through broad search areas with effectiveness while avoiding local optima [6]. Solving optimization issues efficiently is crucial in all domains. However, several conventional optimization techniques—like quadratic programming, gradient optimization, the simplex method, and others—are extremely constrained and difficult to use, making them ineffective for solving real-world problems that are discontinuous and non-differentiable [7]. Because of its straightforward structure, adaptability, and lack of a derivative, the metaheuristic algorithm has gained popularity and is frequently employed in optimization tasks [8]. Swarm intelligence-based optimization algorithms are among the best metaheuristic algorithms. This kind of method simulates various swarm intelligence behaviors in nature and seeks the optimal solution through swarm intelligence; methods such as particle swarm optimization (PSO) [9], gorilla optimization algorithm (GOA) [10], seagull optimization algorithm (SOA) [11], sparrow search algorithm (SSA) [12], dandelion optimization algorithm (DOA) [13], whale optimization algorithm (WOA) [14], golden jackal optimization algorithm (GJO) [15], etc. are all intelligent optimization algorithms based on swarms in nature.

The FOX-inspired optimization algorithm (FOX) is a new intelligent optimization algorithm proposed in 2022 [16]. It has the advantages of simple structure and fast convergence speed, and it has excellent performance in some engineering optimization problems [17]. Nevertheless, there are still some issues that arise when FOX is dealing with high-dimensional difficult optimization problems, namely, the lack of global exploration and the propensity to quickly settle into local extreme values, which can cause the search to stall [18]. The current mainstream strategies of optimization algorithms for solving these problems concentrate on maintaining the optimal value and encouraging the population’s individuals to converge towards it, as well as on enhancing the algorithm’s running behavior logic and initial population position [19,20]. This type of improvement technique increases the algorithm’s capacity for optimization to a certain degree, but it disregards inter-individual information sharing in favor of the global optimal value [21]. Although this technique will surely raise the unnecessary computing cost, it is a novel competitive strategy to compete and learn by categorizing variables hierarchically, such that each variable has its own learning behavior and enriches the population’s variety [22,23].

To sum up the above, this paper adopts a multi-strategy hybrid method to improve the FOX optimization algorithm. Firstly, Tent chaotic mapping is used to initialize the population position and enhance the diversity of the population. Secondly, adaptive weights are introduced to improve the quality of individual locations, optimize search, develop balance parameters, and balance the ability of global search and local search. Then, Levy flight strategy is introduced to update the fox position and greedy strategy is used to select a better position as the new fox position, which makes it possible for the population to jump out of the local optimum. Then, the variable spiral position update strategy is introduced to make the fox position update more flexible, and various position update search paths are developed, and the global and local search of the algorithm are balanced. Finally, ASFFOX, the standard FOX optimization algorithm (FOX), and six other algorithms are compared to the CEC2017 test function set, which verifies the advantages of ASFFOX’s strong optimization ability and jumping out of local optimum. ASFFOX is applied to solving two practical engineering optimization problems, I-beam design problem and reducer design problem, which shows the applicability of ASFFOX in solving engineering optimization problems. The main innovations and contributions of this paper are as follows:

(1) To balance the algorithm’s exploration and development capabilities and accelerate the algorithm’s convergence, adaptive inertia weights are added.

(2) The population is initialized via tent chaotic mapping, and the population diversity is increased by obtaining more ergodic and varied individual beginning locations. To increase the algorithm’s search range, aid in the population’s escape from the local optimum, and enhance convergence accuracy, the inertial weight method is implemented.

(3) The algorithm’s global and local searches are balanced, and the Levy flying method and greedy approach are added to update the fox location. To make the fox position update more flexible, the variable spiral position update technique is used. Different position update search pathways are created, and the algorithm’s global and local searches are balanced.

(4) The ASFFOX algorithm is used to solve engineering optimization problems, increasing the model’s accuracy, resilience, and capacity for generalization. The robustness and excellent global search capability of ASFFOX have been confirmed.

## 2. Fox Optimization Algorithm

Foxes are excellent hunters and may take down animals via top-down or bottom-up attacks. Foxes pursue their prey using incredibly smart techniques in order to live [24,25]. The FOX optimization algorithm mimics how foxes hunt by diving into the snow in search of food [26]. The attractiveness and actions of foxes attempting to find the best prey in the snow serve as the foundation for the main idea. The following are the fundamental steps [27]:

(1) When the ground is covered with a lot of snow, the fox cannot see the prey and starts moving aimlessly in an attempt to find it.

(2) The fox locates the prey by listening to the ultrasonic waves that the prey emits throughout the random stroll. It takes some time to approach its prey after locating the target.

(3) The fox continually locks the precise direction and distance of the prey during the movement by listening to the time difference of the continuous transmission of the prey’s sound waves. The fox then approaches the prey gradually.

(4) The fox determines the angle and direction of the jump that will be required to get the prey after getting close to it.

(5) The fox walks randomly according to the shortest time and the best position.

When the FOX optimization algorithm is running, the population position is first initialized as matrix *X*, and *X* is the matrix of *SearchAgents* row and column *D*, where *SearchAgents* is the population size and *D* is the dimension of the problem [28,29]. Then, the fitness of the search agent is calculated in each iteration. Finally, the best fitness and the best position are obtained by comparing the fitness of rows and rows in the *X* matrix.

In order to balance the exploration and development stages, the random variable *r* is used to assign the possibility proportion of exploration and development. The dynamic variable *a* gradually decreases with the number of iterations, which makes it easy for subsequent iterations to jump out of the local optimum [30].

In the exploration phase, the first step is to calculate the propagation distance of the sound wave signal received by the fox. The range of values for the random variable *p* is [0, 1]. If the random number *p* is greater than 0.18, the fox needs to search for a new location. The distance $Dist\_Fox\_Prey_{it}$ between the fox and its prey, as well as the required jumping height, needs to be calculated. Therefore, at each iteration to it, it is necessary to generate a *D*-dimensional random vector $Time\_s\_T_{it}$ within the range of [0, 1] to represent the sound propagation time. The distance between a fox and its prey is calculated by measuring the propagation speed Sp_S and propagation time $Time\_s\_T_{it}$ of sound in the medium [31].

(1) $$Dist\_S\_T_{it}=Sp\_S\cdot Time\_s\_T_{it}$$

In the formula, $Dist\_S\_T_{it}$ represents the distance of sound propagation. The parameter *it* is the current iteration count. Sp_S is the speed of sound propagation. $Time\_s\_T_{it}$ is a random number within the range of [0, 1] that represents the propagation time of sound.

Based on the time required for sound to propagate between the fox and prey and the optimal position at that time, the value of Sp_S can be determined as shown in the following equation [32].

(2) $$Sp\_S=\frac{BestPosition_{it}}{Time\_s\_T_{it}}$$

wherein, $BestPosition_{it}$ represents the best position of the fox so far. $Time\_s\_T_{it}$ represents the time of sound propagation between foxes and prey.

The distance $Dist\_Fox\_Prey_{it}$ between the fox and its prey is half the distance of sound propagation.

(3) $$Dist\_Fox\_Prey_{it}=0.5Dist\_S\_T_{it}$$

The fox will look for a new location to jump and pounce in order to get its prey after assessing the distance between it and the prey. The fox must so determine its leaping height. The following formula describes the leaping process, which is a parabolic motion:

(4) $$Jump_{it}=\frac{1}{2}gt^{2}$$

In the formula, $Jump_{it}$ represents the jumping height of the fox. The parameter *g* is the acceleration due to gravity, with a value of 9.81. The parameter *t* is the average time required for sound propagation [33].

Use the following two formulas to update the position of the fox.

(5) $$X_{it+1}=Dist\_Fox\_Prey_{it}\cdot Jump_{it}\cdot c_{1}$$

(6) $$X_{it+1}=Dist\_Fox\_Prey_{it}\cdot Jump_{it}\cdot c_{2}$$

In the formula, *c*_1_ and *c*_2_ are divided into position update parameters, with values based on the success rate of fox jumping hunting. Generally, *c*_1_ ∈ [0, 0.18], *c*_2_ ∈ [0.18, 1]. Use a random variable *p* within the range of [0, 1] to determine the position update formula. If *p* > 0.18, update the fox position using Equation (5). But if *p* < 0.18, calculate the new position using Equation (6).

During the fox’s random walk phase, a random search will be conducted based on the best position that has been found so far. In order to ensure that the fox randomly moves to the optimal position, the shortest time control walk is used.

(7) $$tt=\frac{sum\left(Time\_s\_T_{it}(i,:)\right)}{D},MinT=\min(tt)$$

(8) $$a=2\left(it-\frac{1}{Max_{it}}\right)$$

In the formula, the parameter *tt* is the time averaged value for each row. *MinT* is the shortest average time. The parameter *D* is the dimension of the problem being solved. The parameter *a* is an iterative dynamic variable. $Max_{it}$ is the maximum number of iterations [34].

In order to optimize the fox random walk strategy and enhance the global search capability of the FOX algorithm, *MinT* and a variable were used simultaneously. Use the following formula to update the optimal position of the fox.

(9) $$X_{it+1}=BestX_{it}\cdot rand(1,D)\cdot MinT\cdot a$$

## 3. FOX Optimization Algorithm for Adaptive Spiral Flight

### 3.1. Tent Chaotic Mapping Based on Random Variables

The algorithm’s effect is significantly influenced by its beginning value. The method will have a highly positive outcome in the end if the beginning value is near the global optimal solution [35]. The random population initialization used by the majority of swarm intelligence optimization algorithms has several drawbacks, including unequal population distribution, limited population variety, and low population quality. This work uses Tent chaotic mapping, whose equation is as follows [36], to establish the fox population during the population initialization stage of the method in order to overcome this issue.

(10) $$z_{i+1}=\left\{\begin{array}{cc} 2z_{i}+rand(0,1)\times \frac{1}{N} & 0<x_{k}<\frac{1}{2}\\ 2\left(1-z_{i}\right)+rand(0,1)\times \frac{1}{N} & \frac{1}{2}<x_{k}<1 \end{array}\right.$$

Among these, the parameter *N* is the total number of particles in the chaotic sequence, and *z_i+_*_1_ and *z_i_* represent the sequence numbers of the *i*-th and (*i* + 1)-th chaotic sequences, respectively.

According to the characteristics of Tent mapping, the sequence steps for generating chaos in the feasible domain are as follows:

(1) Randomly generate the initial value *z*_0_ in (0, 1); let *i* = 1.

(2) Use Equation (10) for iteration to generate a *z*-sequence, where *i* increases by 1.

(3) If the maximum number of iterations is reached, stop and save the generated *z*-sequence.

Apply the Tent chaotic sequence *z_i+_*_1_ generated by Equation (10) to initialize the position of the fox population, as follows:

(11) $$X_{it}^{new}=(U_{it}-L_{it})z_{i+1}+L_{it}$$

In the formula, *U_it_* and *L_it_* are the upper and lower bounds of the independent variables of the objective function when iterating *it*-th times. $X_{it}^{new}$ is the updated fox population position using Tent chaotic mapping.

### 3.2. Adaptive Inertia Weight

An essential element in the search process is inertia weight, which can keep the participants moving in a specific way, prevent them from arriving at the local best solution, and speed up their movement [37,38]. When the weight is little, the algorithm has high local development ability and can locate the region near the ideal solution; when the weight is big, the algorithm has strong global search capacity. A suitable inertia weight can enhance the algorithm’s global search capability and accelerate the algorithm’s convergence, both of which are crucial for determining the optimal value of the objective function [39]. In this paper, a strategy of adaptively changing the inertia weight according to the number of iterations is proposed. The mathematical expression is as follows:

(12) $$\omega (t)=0.2\cos\left(\frac{\pi }{2}(1-\frac{t}{Max_{it}})\right)$$

where the parameter *t* is the current number of iterations, *Max_it_* is the maximum number of iterations, and the inertia weight *ω*(*t*) changes nonlinearly between [0, 1]. According to the characteristics of cos function, the weight value is small at the beginning of the algorithm, but the optimization speed is faster, and the option value is larger at the end, but the change speed is slow, so the convergence of the algorithm is balanced.

The improved fox jumping hunting position update method is as follows:

(13) $$X_{it+1}=\omega (t)\cdot Dist\_Fox\_Prey_{it}\cdot Jump_{it}\cdot c_{1}$$

(14) $$X_{it+1}=\omega (t)\cdot Dist\_Fox\_Prey_{it}\cdot Jump_{it}\cdot c_{2}$$

In the formula, *c*_1_ and *c*_2_ are divided into position update parameters, with values based on the success rate of fox jumping hunting. Generally, *c*_1_ ∈ [0,0.18], *c*_2_ ∈ [0.18,1]. Use a random the variable *p* within the range of [0, 1] to determine the position update formula. If *p* > 0.18, update the fox position using Equation (13). But if *p* < 0.18, calculate the new position using Equation (14).

By introducing adaptive weights to dynamically adjust the position changes of foxes, the fox’s different position update modes at different times make the algorithm search more flexible. As the number of iterations increases, a single fox converges to the optimal position, and the larger the weight, the faster the individual moves, thus improving the convergence speed of the algorithm.

### 3.3. Levy Flight Mechanism

Aiming at the problem that FOX is easy to fall into local optimum, Levy flight is introduced to update the FOX position [40]. Levy flight is a random flight with alternating search range, which can improve the global search ability of the algorithm, which makes it possible for the algorithm to jump out of the local optimum [41]. The mathematical expression of fox position update is shown in Equation (15):

(15) $$Xl_{it}=X_{it}+l⊕levy(\lambda )$$

In the equation, *Xl_it_* represents the updated position of Levy’s flight. The parameter *l* represents the step size control parameter. *levy* represents the step size that follows the levy distribution, with a value of

(16) $$levy=\frac{\mu }{{\left|\nu \right|}^{\frac{1}{\gamma }}}$$

In the formula, μ and ν follow a normal distribution. $\mu \sim N(0,\sigma_{\mu }^{2})$, $\nu \sim N(0,\sigma_{\nu }^{2})$, and the definitions of $\sigma_{\mu }^{2}$ and $\sigma_{\nu }^{2}$ are as shown in Equation (17):

(17) $$\left\{\begin{array}{c} \sigma_{\mu }={\left[\frac{\Gamma (1+\gamma )\sin(\gamma \pi /2)}{\gamma \Gamma \left((1+\gamma )/2\right)2^{(\gamma +1)/2}}\right]}^{1/\gamma }\\ \sigma_{\upsilon }=1 \end{array}\right.$$

where, γ is 1.5.

After the fox position is updated by Levy flight, the fox position updated by Levy flight is not necessarily a better position due to the random flight characteristics of Levy flight, so the greedy strategy is adopted to choose whether to keep the original position or the new position, and the selection formula is shown in Equation (18):

(18) $$X_{it}=\left\{\begin{array}{c} X_{it},fitness\left(X_{it}\right)\leq fitness\left(Xl_{it}\right)\\ Xl_{it},fitness\left(X_{it}\right)>fitness\left(Xl_{it}\right) \end{array}\right.$$

In the formula, *fitness*(*X_it_*) and *fitness*(*Xl_it_*) respectively represent the fitness values corresponding to the original fox position of the algorithm and the fitness values corresponding to the fox position updated by Levy flight.

### 3.4. Variable Spiral Search Strategy

The fox will randomly seek in the random walk stage in accordance with the best location that has been identified thus far, which results in a monotonous search strategy and may fall into the local optimum, diminishing the algorithm’s search capability [42,43]. A variable spiral position update method is proposed to make the fox position update more flexible, develop different position update search pathways, and balance the algorithm’s global and local search, all of which are inspired by the spiral operation of the whale optimization algorithm [44]. The following is the formula for the FOX variable helix position updating strategy:

(19) $$X_{it+1}=e^{zl}\cdot \cos(2\pi l)\cdot BestX_{it}\cdot rand(1,D)\cdot MinT\cdot a$$

(20) $$z=e^{k\cdot \cos\left(\pi \cdot (1-(it/Max_{it}))\right)}$$

where, the parameter *k* is the coefficient of variation, *k* = 5. The parameter *l* is a uniformly distributed random number of [−1, 1]. With the range of fox position updates from large to small, more high-quality solutions are found in the early stage, and the late optimization reduces the increase of idle work, thus improving the global optimal search performance of the algorithm. At the same time, according to the characteristics of spiral, the optimization accuracy of the algorithm is improved to a certain extent.

### 3.5. Time Complexity Analysis

The time complexity of the algorithm is mainly related to the number of individual fox populations (*SearchAgents*), individual dimensions (*D*), and the number of algorithm iterations (*Max_it_*). The time complexity of FOX is *O*(SearchAgents*D*Max_it_). The time complexity of introducing Tent chaotic mapping to initialize the population is *O*(SearchAgents*D). Introducing adaptive inertia weight and variable spiral search strategy does not change the time complexity of the algorithm. Introducing Levy flight strategy to update the fox position has a time complexity of *O*(SearchAgents*D), so the time complexity within the iteration loop is *O*(SearchAgents*D). Therefore, the time complexity of ASFFOX is *O*(SearchAgents*D*Max_it_), which is the same as FOX’s time complexity. The improvement does not increase ASFFOX’s time complexity.

### 3.6. Algorithm Flowchart and Pseudocode

The pseudocode of the ASFFOX algorithm proposed in this article is shown in Algorithm 1.
**Algorithm 1** Pseudo code of ASFFOX algorithm1: Initialize the population according to Equation (11); 2: **While *it* < *Max_it_***
3:     Initialize *Dist_S_T,Sp_S,Time_S_T,BestX,Dist_Fox_Prey,Jump,MinT,a,BestFitness,ω*(*t*); 4:     Calculate the *fitness* of each search agent 5:     Select *BestX* and *BestFitness* among the population(*X*) in each iteration; 6:     **If1** *fitness*(*X_it_*) > *fitness*(*X_it+_*_1_) 7:        *BestFitness* = *fitness*(*X_it+_*_1_); 8:        *BestX* = *X*(*i*,:); 9:     **Endif1**
10:    **If2** *r ≥* 0.5 11:       **If3** *p >* 0.18 12:           Initialize time randomly; 13:           Calculate Distance_Sound_travels using Equation (1); 14:           Calculate *Sp_S* from Equation (2); 15:           Calculate distance from fox to prey using Equation (3); 16:           *Tt* = average time; 17:           *T = Tt*/2; 18:           Calculate jump using Equation (4) 19:           Find *X_it_*_+1_ using Equation (13); 20:       **Elseif** *p ≤* 0.18 21:           Initialize time randomly; 22:           Calculate Distance_Sound_travels using Equation (1) 23:           Calculate *Sp_S* from Equation (2) 24:           Calculate distance from fox to prey using Equation (3) 25:           Tt = average time; 26:           *T = Tt*/2; 27:           Calculate jump using Equation (4) 28:           Find *X_it+_*_1_ using Equation (14); 29:       **EndIf3**
30:    else 31:       Find *MinT* using Equation (7); 32:       Explore *X_it+_*_1_ using Equations (19) and (20); 33:    **EndIf2**
34:    Explore *X_it+_*_1_ using Equations (15)–(18); 35:    Check and amend the position if it goes beyond the limits; 36:    Evaluate search agents by their fitness; 37:    Update *BestX;*
38:    *it = it +* 1; 39: **End while**
40: return *BestX* and *BestFitness*

## 4. Simulation Experiment Results and Analysis

In order to verify the superiority and feasibility of the improved ASFFOX algorithm, the proposed ASFFOX algorithm is compared with other classical metaheuristic algorithms, such as gray wolf optimization algorithm (GWO) [45], Harris hawks optimization (HHO) [46], whale optimization algorithm (WOA) [47], dung beetle optimization algorithm (DBO) [48], crayfish optimization algorithm (COA) [49], osprey optimization algorithm (OOA) [50], and FOX optimization algorithm (FOX), respectively. The test function details are shown in Table 1, where F1 to F3 are unimodal functions, F4 to F10 are multimodal functions, F11 to F20 are mixed functions, and F21 to F30 are composition functions. Different test functions are used to test different attributes. Since F2 is unstable when using MATLAB software to conduct experiments, 29 test functions except F2 are used in this experiment.

### 4.1. Experimental Parameter Settings

The Intel (R) Core (TM) i9-13900H @ 2.60 GHz CPU serves as the experimental environment and MATLAB R2022b as the experimental platform, and the Windows 11 operating system completes the numerical experiment. The comparison’s other algorithmic control settings all agree with the relevant original references.

To ensure equity, every algorithm is assigned a population size of *N* = 30, undergoes 30,000 assessment cycles, and is executed 30 times on its own. To demonstrate the correctness and stability of the algorithm, the ideal value, standard deviation, and average value of the solution results for each test function are noted. Experiments from two dimensions—30 and 50—will be carried out in this research to confirm the efficacy of the ASFFOX improvement technique.

### 4.2. Comparative Analysis of ASFFOX and Other Metaheuristic Algorithms

**Figure 1:**
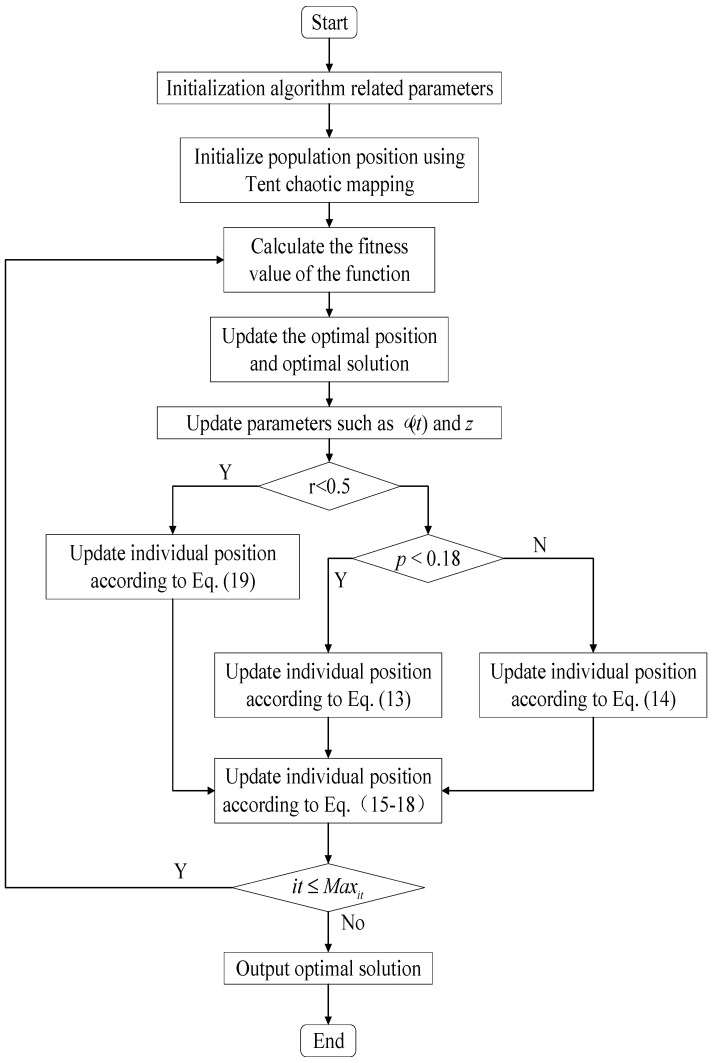
The flowchart of ASFFOX algorithm.

**Figure 2:**
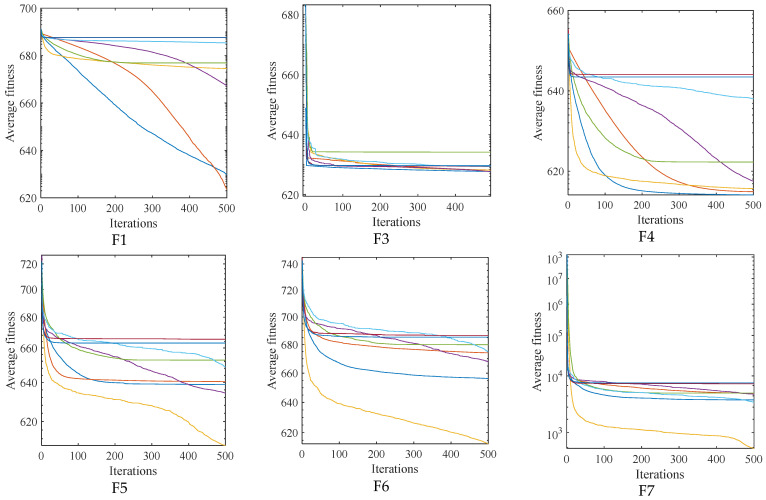

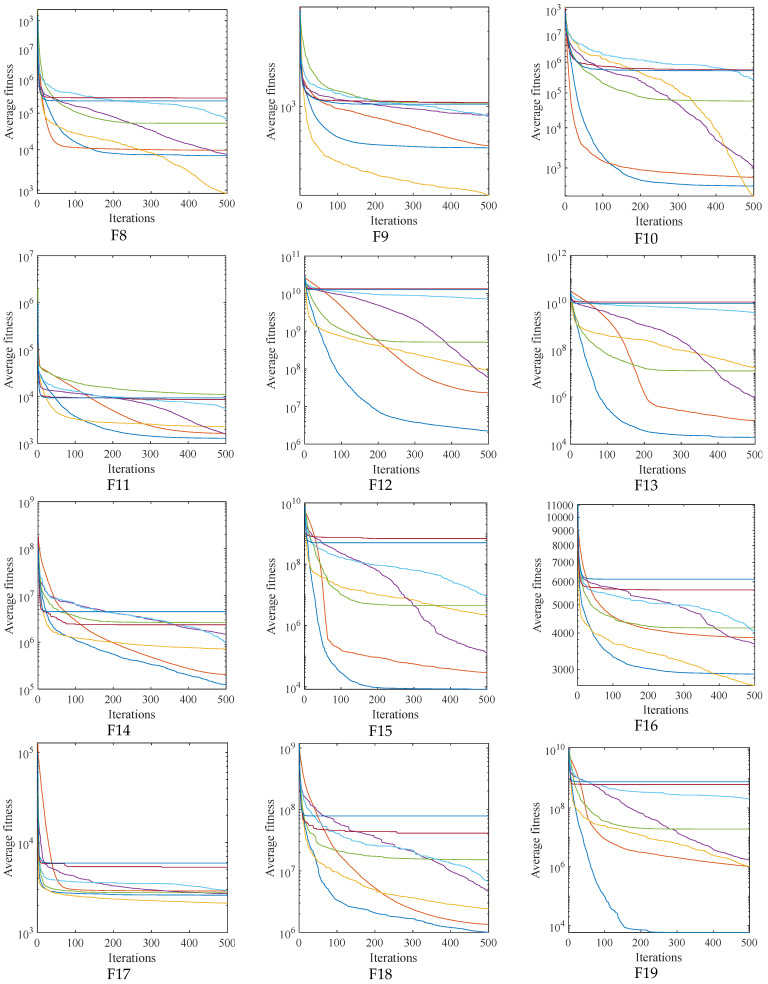

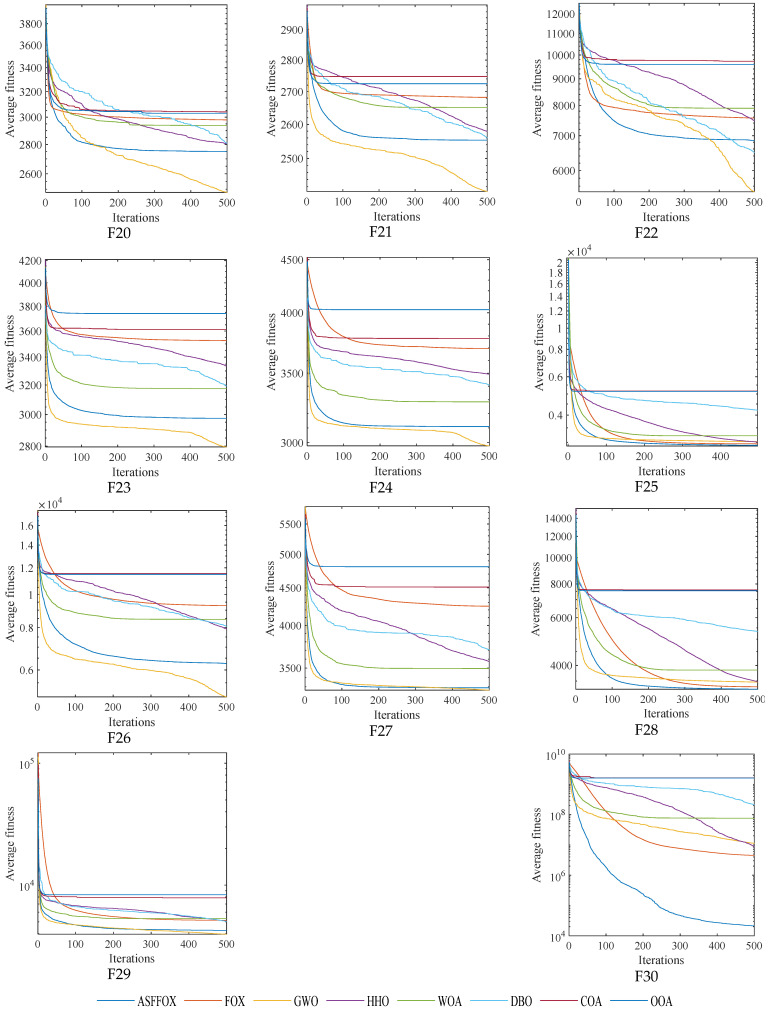
The iterative convergence curves of 8 algorithms (D = 30).

To verify the optimization performance of the ASFFOX algorithm, the proposed ASFFOX algorithm and traditional metaheuristic algorithm were simulated on the CEC2017 test set function. The experimental dimension was 30, and the results of 30 independent runs are shown in Table 2. The average convergence curve of the iteration is shown in Figure 1, and the data box plot is shown in Figure 2.

**Figure 3:**
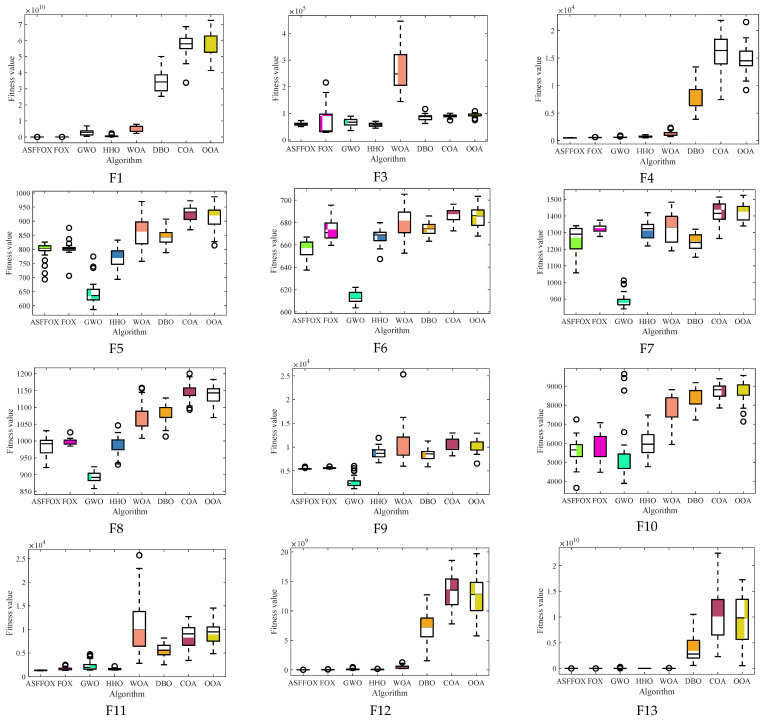

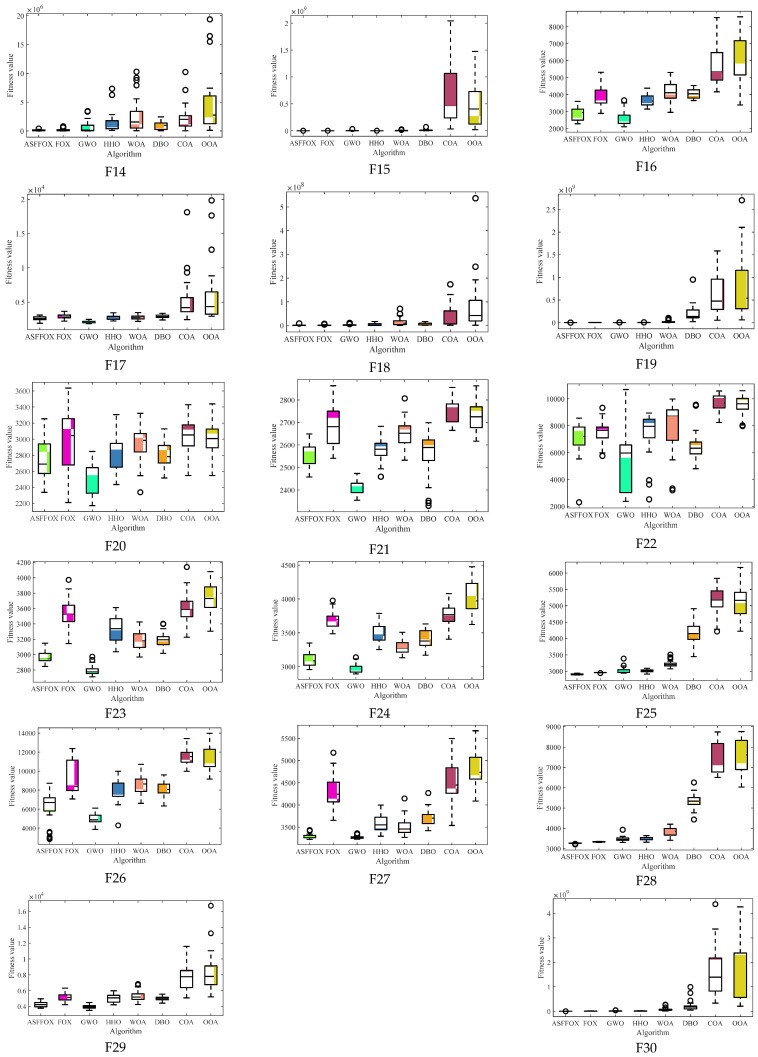
The box plots of iterative data distributions (D = 30).

By analyzing the data in Figure 2, it is evident that FOX finds the best value when solving the unimodal functions F1 and F3, and that the improved algorithm in this paper finds the best value at F4 and F10, with the remaining values only surpassed by GWO, and that its stability is also at the forefront when solving the multi-modal functions F4 to F10; ASFFOX finds the best values on 11 functions by solving the mixed functions F11 to F20 and the combination functions F21 to F30. With the exception of F20 and F21, the solved values exhibit good standard deviation on the majority of functions and rank second only to the optimum values, demonstrating the algorithm’s stability and strong solution accuracy. With the exception of the F14 function, which ranks second in the comparison algorithm, none of the 29 functions have a lower accuracy than FOX. This indicates that the enhanced FOX optimization algorithm is capable of searching the exploration space more thoroughly and effectively, and it also possesses strong global optimization and local exploration capabilities. A box plot of the data distribution for the various methods in the article is shown in Figure 3. It is evident that the technique suggested in this work performs best in most circumstances by having the narrowest range of solutions for determining the function’s minimal value.

Figure 2 and Figure 3 show that the ASFFOX technique takes less iterations to get the same accuracy while solving multimodal functions, indicating a quicker rate of convergence for the enhanced approach. The convergence curve of ASFFOX leaps a lot throughout the multi-peak test function optimization process, indicating that it is more adept than FOX in jumping out of the local optimum and exploring the world. ASFFOX consistently performs better than the comparison algorithm in the upper, lower, upper quartile, lower quartile, and median of the majority of test functions, as shown by the box plot in Figure 3.

### 4.3. High Dimensional Benchmark Function Testing

**Figure 4:**
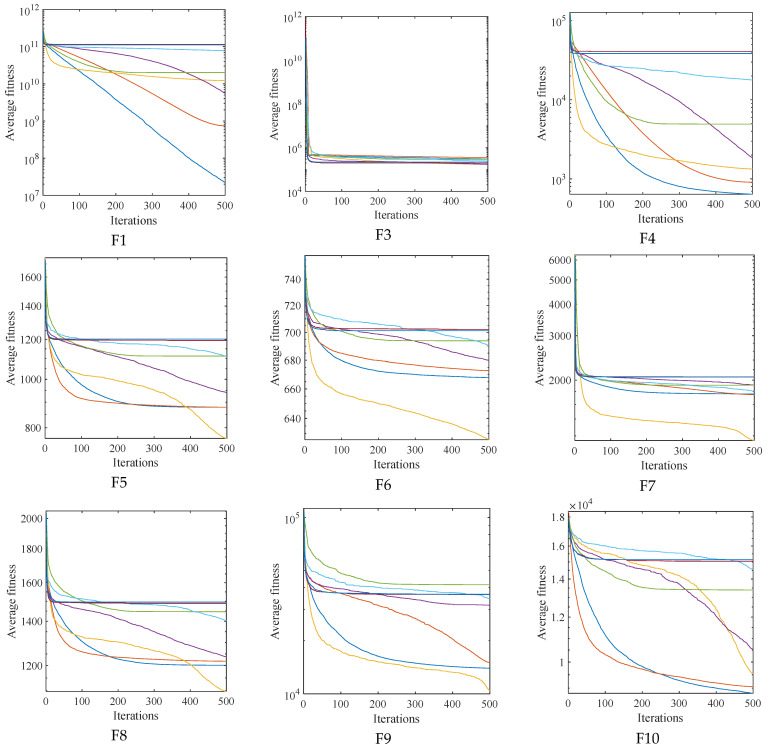

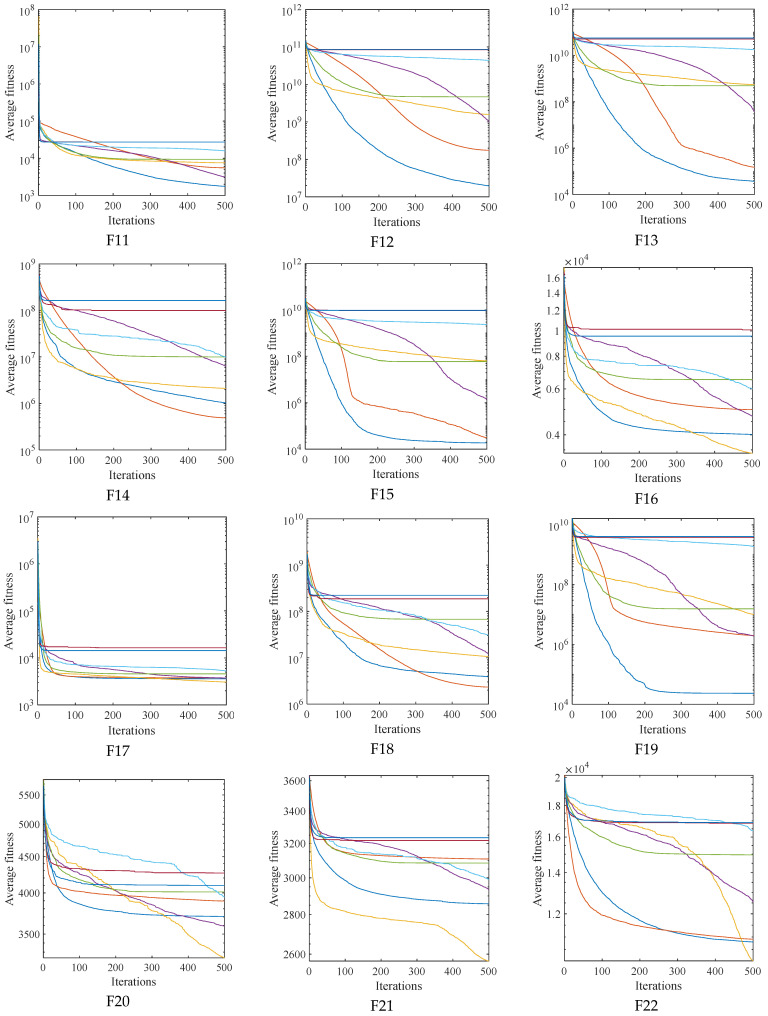

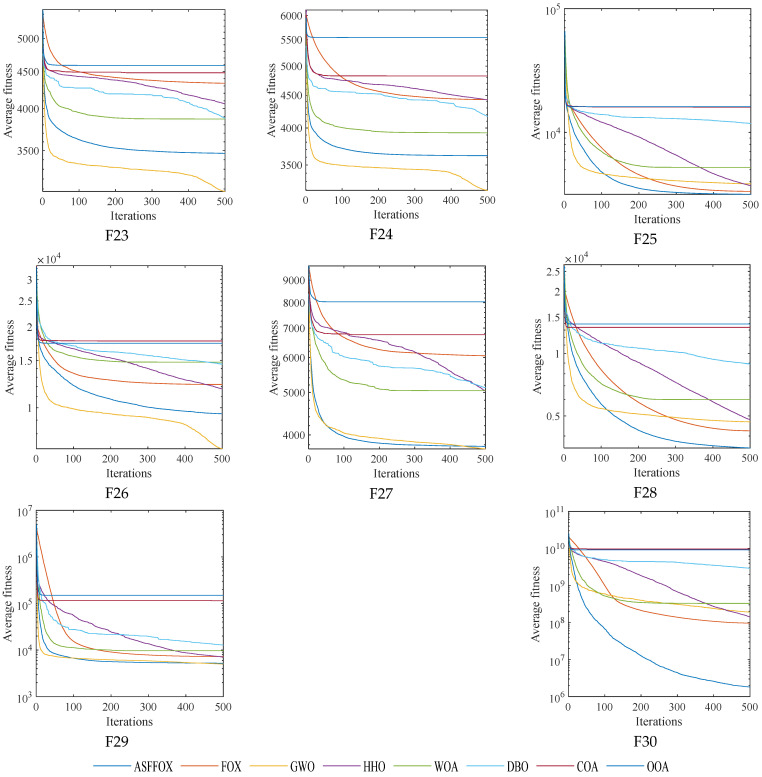
The iterative convergence curves of 8 algorithms (D = 50).

**Figure 5:**
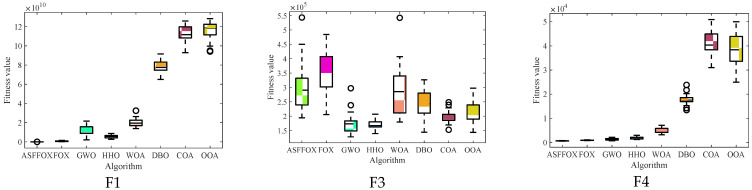

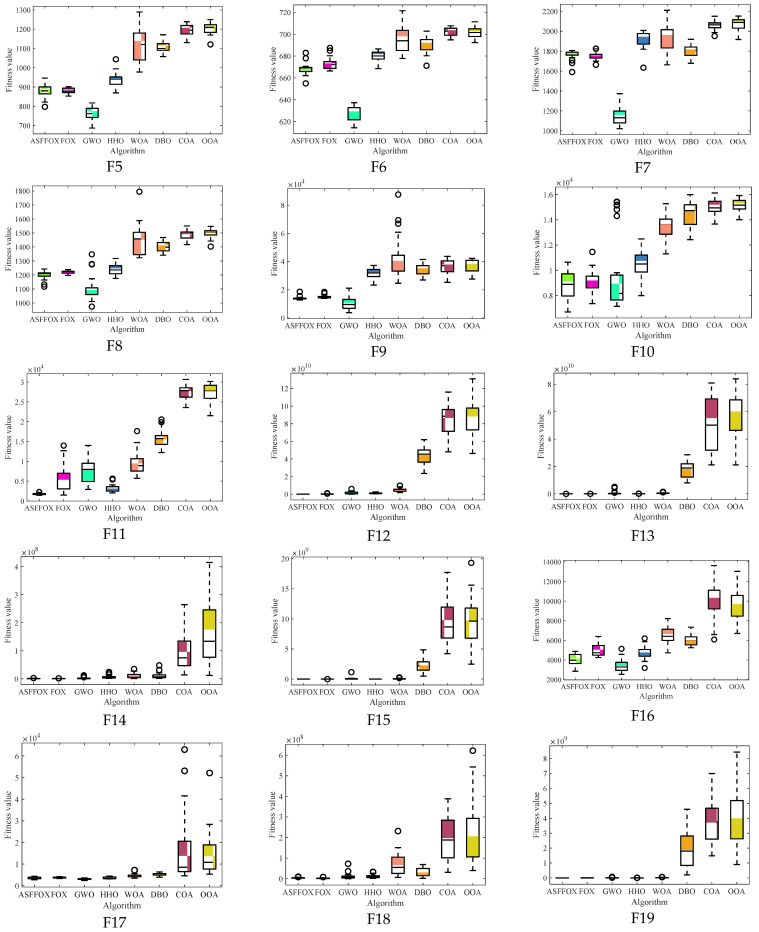

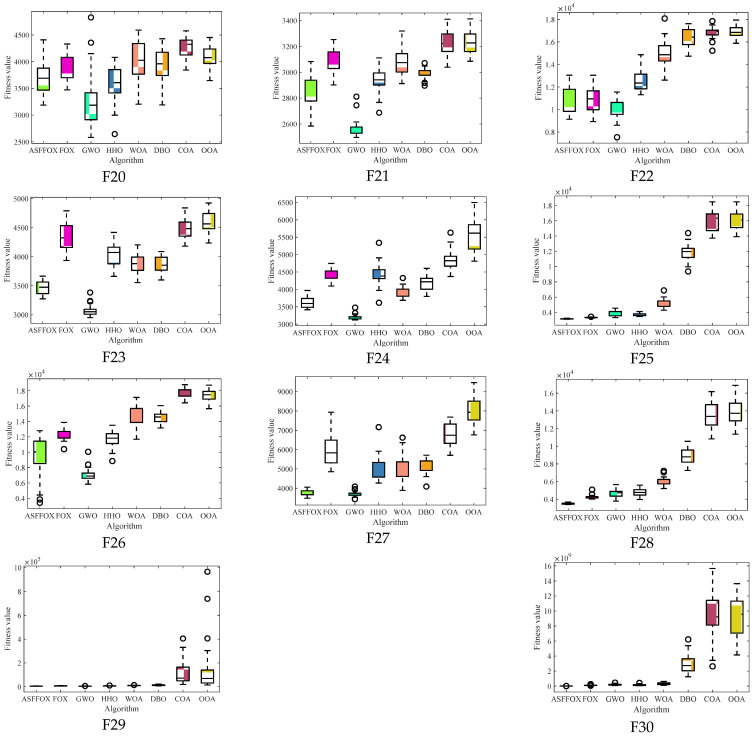
The box plots of iterative data distributions (D = 50).

This part modifies the test dimension to 50 dimensions and evaluates the optimization performance of each method in the CEC2017 test function in order to investigate the optimization performance of the upgraded algorithm on the high-dimensional benchmark function in more detail. Similar to this, there are 500 iterations, 30 separate runs of the experiment, and a population size of 30. Table 3 displays the detailed experimental findings. Figure 4 and Figure 5 record the data box diagram and the average convergence curve of the iterations, respectively. According to the experimental results, ASFFOX’s anti-stagnation ability is more pronounced and its capacity to escape local extremum is significantly better than that of the original algorithm. The ASFFOX method continues to outperform the comparison algorithm in terms of optimization performance over 50 dimensions, and it remains highly competitive in terms of convergence accuracy and stability.

### 4.4. Wilcoxon Rank Sum Test

Merely evaluating an algorithm’s performance only on the basis of its mean error and standard deviation is insufficient. To confirm whether there is a substantial difference between ASFFOX and the comparison algorithm’s optimization outputs, more statistical test techniques are required. The experiment in this part uses the Wilcoxon rank sum test, and the significance assessment index *p* is set at 5%. The optimization outcomes of ASFFOX and GWO, HHO, WOA, DBO, COA, OOA, and FOX algorithms are determined to be significantly different based on a comparison of the average error values of each algorithm ran separately for 30 tests. Table 4 displays the statistical findings. A statistically significant difference exists between ASFFOX and the comparison algorithm in the present function when *p* < 0.05. *p* > 0.05 indicates that there is not a discernible difference between the comparison algorithm in the present function and ASFFOX. When *p* is *NaN*, there is no difference between the two methods and the significance test between them cannot be conducted. If ASFFOX and the comparison algorithm do not significantly vary in a particular function, then the two methods perform equally. The convergence accuracy of the two algorithms in this function is compared to assess performance in the event that there is a notable discrepancy between ASFFOX and the comparison algorithm.

From the statistical results, it can be seen that the *p* values of most rank sum tests are less than 0.05, which indicates that there is a significant difference between the optimization results of the ASFFOX algorithm and the seven comparison algorithms. The experimental results in this section further verify that the experiments in Section 4.2 and Section 4.3 are reliable, showing that the four improved strategies introduced by the FOX algorithm can effectively balance the global exploration and local development capabilities of the algorithm, avoid the algorithm falling into local optimum, and make the ASFFOX algorithm have better performance.

### 4.5. Application of Engineering Optimization Problems

To test the feasibility of the improved algorithm in solving practical engineering optimization problems, two engineering optimization problems were selected and solved using eight algorithms.

#### 4.5.1. Optimization Problem of I-Beam Design

Engineering optimization problem 1 is a design problem for I-beams. Its purpose is to minimize the vertical deflection of the beam to the greatest extent possible. It simultaneously satisfies the cross-sectional area and stress constraints under a given load [51]. This problem contains four variables and two constraints, and the mathematical description of the problem is as follows:

Objective function:

(21) $$f(X)=\frac{5000}{x_{3}{\left(x_{2}-2x_{4}\right)}^{3}/12+(x_{1}x_{4}^{3}/6)+2x_{2}x_{4}{\left(x_{2}-x_{4}/2\right)}^{2}}$$

Constraint condition:

(22) $$g_{1}(X)=2x_{1}x_{3}+x_{3}(x_{2}-2x_{4})\leq 300$$

(23) $$g_{2}(X)=\frac{18x_{2}\times 10^{4}}{x_{3}{\left(x_{2}-2x_{4}\right)}^{3}+2x_{1}x_{3}\left(4x_{4}^{2}+3x_{2}\left(x_{2}-2x_{4}\right)\right)}+\frac{15x_{1}\times 10^{3}}{\left(x_{2}-2x_{4}\right)x_{3}^{2}+2x_{3}x_{1}^{3}}\leq 56$$

Value range:

(24) $$10\leq x_{1}\leq 50$$

(25) $$10\leq x_{2}\leq 80$$

(26) $$0.9\leq x_{3}\leq 4$$

(27) $$0.9\leq x_{4}\leq 4$$

**Figure 6:**
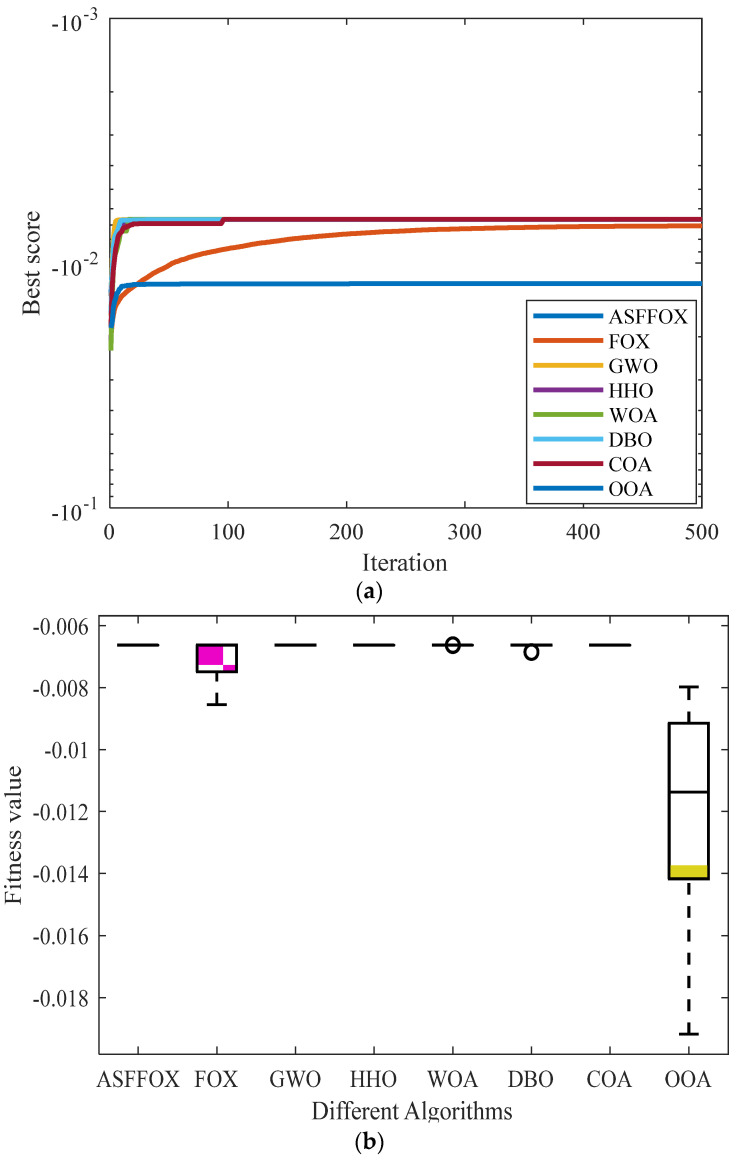
Iterative curves and box plots for design issues with I-beams problem. (**a**) Iterative convergence curve. (**b**) Iterative data distribution boxplot.

Select the ASFFOX algorithm and seven algorithms including GWO, HHO, WOA, DBO, COA, OOA, and FOX to jointly solve engineering optimization problem 1, with an evaluation frequency of T = 1500 times; the population size is *N* = 30. Each algorithm runs independently 10 times, and the solution results are recorded in Table 5. The average convergence curve of iterations and the plotted data box plot are shown in Figure 6. From the data in Table 5, it can be analyzed that ASFFOX exhibits the best mean, standard deviation, and convergence accuracy among all the algorithms involved in the calculation, proving that the algorithm has good convergence ability and runs in a relatively stable manner. From the iterative curve and box plot in Figure 6, we can see that ASFFOX exhibits good convergence and stability in solving I-beam design problems.

#### 4.5.2. Weight Minimization of a Speed Reducer

Engineering optimization problem 2 is a gearbox design problem. The design problem of a reducer is a typical mechanical optimization problem, with the goal of minimizing the weight of the reducer to facilitate the normal operation of the propeller and engine [52]. This problem involves seven decision variables. The mathematical description of the problem is as follows:

Objective function:

(28) $$\begin{array}{ll} \min f(x)= & 0.7854x_{1}x_{2}^{2}(3.3333x_{3}^{2}+14.9334x_{3}-43.0934)-1.508x_{1}(x_{6}^{3}+x_{7}^{2})\\ & +7.4777(x_{6}^{3}+x_{7}^{3})+0.7854(x_{4}x_{6}^{2}+x_{5}x_{7}^{2}) \end{array}$$

Constraint condition:

(29) $$g_{1}(x)=\frac{27}{x_{1}x_{2}^{2}x_{3}}-1\leq 0$$

(30) $$g_{2}(x)=\frac{397.5}{x_{1}x_{2}^{2}x_{3}}-1\leq 0$$

(31) $$g_{3}(x)=\frac{1.93x_{5}^{3}}{x_{1}x_{6}^{4}x_{3}}-1\leq 0$$

(32) $$g_{4}(x)=\frac{1.93x_{5}^{3}}{x_{2}x_{7}^{4}x_{3}}-1\leq 0$$

(33) $$g_{5}(x)=\frac{{\left[{\left(745(x_{4}/x_{2}x_{3})\right)}^{2}+16.9\times 10^{6}\right]}^{1/2}}{110x_{6}^{3}}-1\leq 0$$

(34) $$g_{6}(x)=\frac{{\left[{\left(745(x_{5}/x_{2}x_{3})\right)}^{2}+157.5\times 10^{6}\right]}^{1/2}}{85x_{7}^{3}}-1\leq 0$$

(35) $$g_{7}(x)=\frac{x_{2}x_{3}}{40}-1\leq 0$$

(36) $$g_{8}(x)=\frac{5x_{2}}{x_{1}}-1\leq 0$$

(37) $$g_{9}(x)=\frac{5x_{1}}{12x_{2}}-1\leq 0$$

(38) $$g_{10}(x)=\frac{1.5x_{6}+1.9}{x_{4}}-1\leq 0$$

(39) $$g_{11}(x)=\frac{1.1x_{7}+1.9}{x_{5}}-1\leq 0$$

Value range:

(40) $$2.6\leq x_{1}\leq 3.6;$$

(41) $$0.7\leq x_{2}\leq 0.8;$$

(42) $$17\leq x_{3}\leq 28;$$

(43) $$7.3\leq x_{4};$$

(44) $$x_{5}\leq 8.3;$$

(45) $$2.9\leq x_{6}\leq 3.9;$$

(46) $$5.0\leq x_{7}\leq 5.5;$$

**Figure 7:**
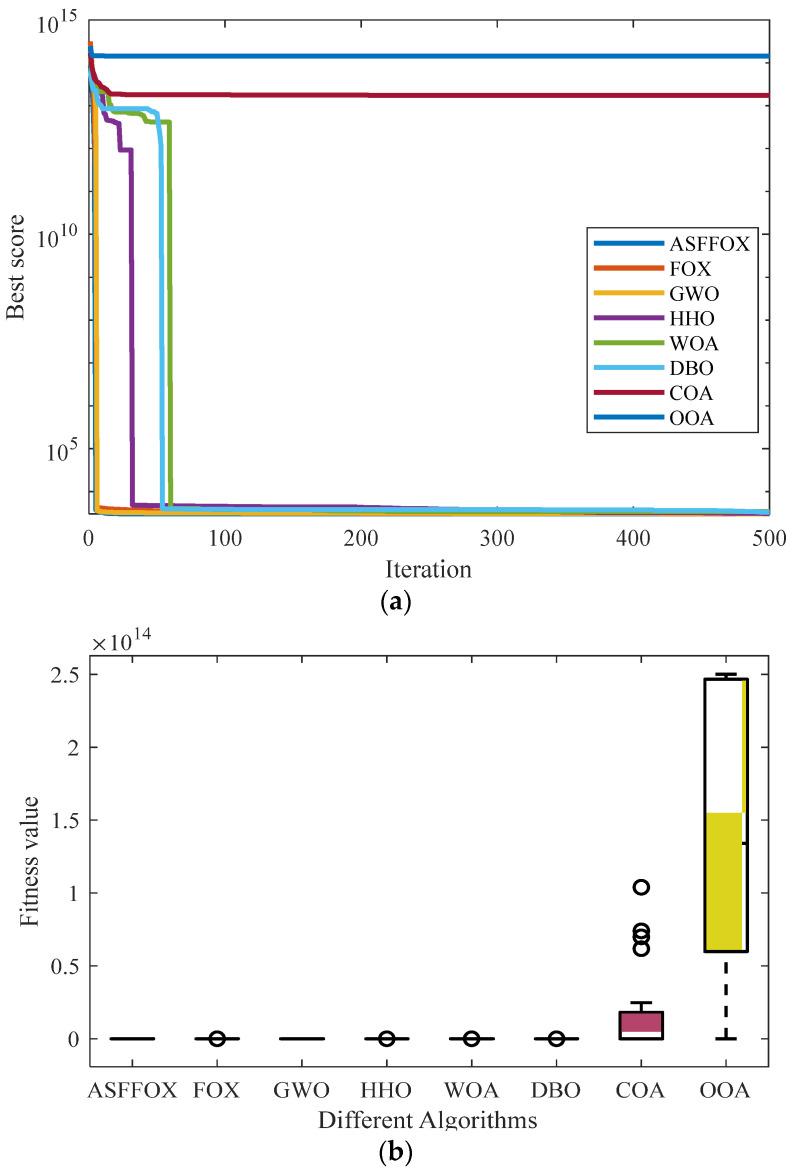
Iterative curves and box plots of speed reducer optimization design problem. (**a**) Iterative convergence curve. (**b**) Iterative data distribution boxplot.

Table 6 shows the experimental results obtained on eight algorithms for the design problem of the reducer. The average convergence curve of the iteration and the data box plot are shown in Figure 7. From Table 6, it can be seen that the ASFFOX algorithm obtains the minimum reducer weight of 2994.4, and its optimal value, standard deviation, and mean are all optimal, proving its good solving accuracy and stability. From the iterative curve and box plot in Figure 7, it can be seen that ASFFOX has better stability and convergence speed than other algorithms, indicating that the ASFFOX algorithm has good optimization ability and stability.

## 5. Conclusions

This study presents an adaptive spiral flying fox optimization method, which aims to address the issues of weak individual ergodicity and easy fall into local optimum of the FOX algorithm. The introduction of tent chaotic mapping yields an initial population of excellent quality. The fox jumping and hunting technique is altered, and the FOX algorithm’s convergence speed and accuracy are enhanced, by raising the inertia weight. The use of Levy flight and variable spiral search method aims to enhance the precision of the FOX algorithm and its capacity to exit the local optimum.

The application ability of the algorithm is tested using two real-world engineering challenges and a multi-dimensional comparison experiment on the CEC2017 test set between the ASFFOX method and seven conventional swarm intelligence algorithms. In contrast, the ASFFOX method has strong computational stability and has a quicker rate of convergence, higher accuracy of convergence, and superior capacity to break out of the local optimum. The outcomes confirm the algorithm’s efficacy. In the following phase, we will carry out a more thorough investigation, strengthen the algorithm’s capacity for optimization in fixed low dimensions, and consistently increase its adaptability to more effectively address a range of real-world optimization issues. Simultaneously, we will keep refining the Fox optimization algorithm’s optimization mechanism, continuously boosting its solution efficiency, increasing its suitability for handling engineering application problems, and using it to solve optimization issues in a wider range of domains.

## Figures and Tables

**Table 1:** CEC 2017 Test Functions.

	No.	Functions	*Fi = Fi*(*x*)
Unimodal Functions	1	Shifted and Rotated Bent Cigar Function	100
	2	Shifted and Rotated Sum of Different Power Function *	200
	3	Shifted and Rotated Zakharov Function	300
Simple Multimodal Functions	4	Shifted and Rotated Rosenbrock’s Function	400
	5	Shifted and Rotated Rastrigin’s Function	500
	6	Shifted and Rotated Expanded Scaffer’s F6 Function	600
	7	Shifted and Rotated Lunacek Bi_Rastrigin Function	700
	8	Shifted and Rotated Non-Continuous Rastrigin’s Function	800
	9	Shifted and Rotated Levy Function	900
	10	Shifted and Rotated Schwefel’s Function	1000
Hybrid Functions	11	Hybrid Function 1 (*N* = 3)	1100
	12	Hybrid Function 2 (*N* = 3)	1200
	13	Hybrid Function 3 (*N* = 3)	1300
	14	Hybrid Function 4 (*N* = 4)	1400
	15	Hybrid Function 5 (*N* = 4)	1500
	16	Hybrid Function 6 (*N* = 4)	1600
	17	Hybrid Function 6 (*N* = 5)	1700
	18	Hybrid Function 6 (*N* = 5)	1800
	19	Hybrid Function 6 (*N* = 5)	1900
	20	Hybrid Function 6 (*N* = 6)	2000
Composition Functions	21	Composition Function 1 (*N* = 3)	2100
	22	Composition Function 2 (*N* = 3)	2200
	23	Composition Function 3 (*N* = 4)	2300
	24	Composition Function 4 (*N* = 4)	2400
	25	Composition Function 5 (*N* = 5)	2500
	26	Composition Function 6 (*N* = 5)	2600
	27	Composition Function 7 (*N* = 6)	2700
	28	Composition Function 8 (*N* = 6)	2800
	29	Composition Function 9 (*N* = 3)	2900
	30	Composition Function 10 (*N* = 3)	3000
Search Range: [−100, 100]			

**Table 2:** The test results of the 8 algorithms improved by different strategies (D = 30).

		ASFFOX	FOX	GWO	HHO	WOA	DBO	COA	OOA
F1	min	1.52 × 10^4^	1.19 × 10^4^	3.53 × 10^8^	9.25 × 10^7^	2.26 × 10^9^	2.53 × 10^10^	3.38 × 10^10^	4.14 × 10^10^
F1	std	7.55 × 10^4^	9.01 × 10^3^	1.64 × 10^9^	4.48 × 10^8^	1.65 × 10^9^	6.42 × 10^9^	6.76 × 10^9^	7.83 × 10^9^
F1	avg	9.99 × 10^4^	2.32 × 10^4^	2.85 × 10^9^	5.34 × 10^8^	4.89 × 10^9^	3.43 × 10^10^	5.73 × 10^10^	5.82 × 10^10^
F3	min	5.02 × 10^4^	2.79 × 10^4^	3.53 × 10^4^	4.39 × 10^4^	1.44 × 10^5^	6.17 × 10^4^	7.37 × 10^4^	7.44 × 10^4^
F3	std	5.07 × 10^3^	5.37 × 10^4^	1.31 × 10^4^	6.92 × 10^3^	6.91 × 10^4^	1.12 × 10^4^	6.25 × 10^3^	6.73 × 10^3^
F3	avg	5.92 × 10^4^	6.70 × 10^4^	6.62 × 10^4^	5.77 × 10^4^	2.63 × 10^5^	8.56 × 10^4^	9.01 × 10^4^	9.30 × 10^4^
F4	min	4.65 × 10^2^	5.21 × 10^2^	5.19 × 10^2^	5.06 × 10^2^	7.47 × 10^2^	3.88 × 10^3^	7.45 × 10^3^	9.18 × 10^3^
F4	std	2.44 × 10^1^	3.14 × 10^1^	8.17 × 10^1^	1.41 × 10^2^	4.08 × 10^2^	2.23 × 10^3^	3.49 × 10^3^	2.53 × 10^3^
F4	avg	5.08 × 10^2^	5.58 × 10^2^	6.09 × 10^2^	7.55 × 10^2^	1.30 × 10^3^	7.93 × 10^3^	1.60 × 10^4^	1.48 × 10^4^
F5	min	6.93 × 10^2^	7.06 × 10^2^	5.86 × 10^2^	6.94 × 10^2^	7.57 × 10^2^	7.88 × 10^2^	8.69 × 10^2^	8.14 × 10^2^
F5	std	3.14 × 10^1^	2.43 × 10^1^	4.18 × 10^1^	3.27 × 10^1^	6.59 × 10^1^	2.64 × 10^1^	2.94 × 10^1^	4.08 × 10^1^
F5	avg	7.94 × 10^2^	8.02 × 10^2^	6.45 × 10^2^	7.71 × 10^2^	8.63 × 10^2^	8.44 × 10^2^	9.27 × 10^2^	9.15 × 10^2^
F6	min	6.37 × 10^2^	6.60 × 10^2^	6.04 × 10^2^	6.47 × 10^2^	6.53 × 10^2^	6.63 × 10^2^	6.73 × 10^2^	6.68 × 10^2^
F6	std	7.99 × 10^0^	9.41 × 10^0^	4.96 × 10^0^	6.64 × 10^0^	1.18 × 10^1^	5.84 × 10^0^	5.99 × 10^0^	8.47 × 10^0^
F6	avg	6.56 × 10^2^	6.74 × 10^2^	6.13 × 10^2^	6.68 × 10^2^	6.80 × 10^2^	6.75 × 10^2^	6.86 × 10^2^	6.85 × 10^2^
F7	min	1.06 × 10^3^	1.28 × 10^3^	8.42 × 10^2^	1.22 × 10^3^	1.19 × 10^3^	1.15 × 10^3^	1.26 × 10^3^	1.34 × 10^3^
F7	std	7.44 × 10^1^	2.34 × 10^1^	4.31 × 10^1^	5.10 × 10^1^	8.12 × 10^1^	4.80 × 10^1^	6.38 × 10^1^	4.86 × 10^1^
F7	avg	1.26 × 10^3^	1.32 × 10^3^	8.94 × 10^2^	1.32 × 10^3^	1.33 × 10^3^	1.24 × 10^3^	1.42 × 10^3^	1.43 × 10^3^
F8	min	9.21 × 10^2^	9.85 × 10^2^	8.59 × 10^2^	9.30 × 10^2^	1.01 × 10^3^	1.01 × 10^3^	1.09 × 10^3^	1.07 × 10^3^
F8	std	2.75 × 10^1^	7.99 × 10^0^	1.67 × 10^1^	2.73 × 10^1^	4.00 × 10^1^	2.65 × 10^1^	2.43 × 10^1^	2.97 × 10^1^
F8	avg	9.84 × 10^2^	9.99 × 10^2^	8.92 × 10^2^	9.88 × 10^2^	1.07 × 10^3^	1.08 × 10^3^	1.14 × 10^3^	1.14 × 10^3^
F9	min	5.32 × 10^3^	5.41 × 10^3^	1.25 × 10^3^	6.71 × 10^3^	5.99 × 10^3^	5.85 × 10^3^	8.18 × 10^3^	6.55 × 10^3^
F9	std	1.09 × 10^2^	1.40 × 10^2^	1.26 × 10^3^	1.13 × 10^3^	3.77 × 10^3^	1.23 × 10^3^	1.39 × 10^3^	1.38 × 10^3^
F9	avg	5.45 × 10^3^	5.62 × 10^3^	2.72 × 10^3^	8.76 × 10^3^	1.05 × 10^4^	8.52 × 10^3^	1.06 × 10^4^	1.03 × 10^4^
F10	min	3.65 × 10^3^	4.47 × 10^3^	3.88 × 10^3^	4.77 × 10^3^	5.94 × 10^3^	7.22 × 10^3^	7.85 × 10^3^	7.14 × 10^3^
F10	std	6.91 × 10^2^	6.95 × 10^2^	1.44 × 10^3^	6.40 × 10^2^	6.62 × 10^2^	5.03 × 10^2^	4.12 × 10^2^	5.49 × 10^2^
F10	avg	5.60 × 10^3^	5.79 × 10^3^	5.39 × 10^3^	6.01 × 10^3^	7.75 × 10^3^	8.42 × 10^3^	8.74 × 10^3^	8.71 × 10^3^
F11	min	1.21 × 10^3^	1.28 × 10^3^	1.39 × 10^3^	1.36 × 10^3^	2.79 × 10^3^	2.48 × 10^3^	3.40 × 10^3^	4.83 × 10^3^
F11	std	4.53 × 10^1^	2.91 × 10^2^	9.60 × 10^2^	1.75 × 10^2^	6.04 × 10^3^	1.40 × 10^3^	2.45 × 10^3^	2.47 × 10^3^
F11	avg	1.29 × 10^3^	1.65 × 10^3^	2.27 × 10^3^	1.59 × 10^3^	1.12 × 10^4^	5.60 × 10^3^	8.68 × 10^3^	9.38 × 10^3^
F12	min	2.56 × 10^5^	1.45 × 10^6^	7.85 × 10^6^	5.93 × 10^6^	2.17 × 10^8^	1.55 × 10^9^	7.80 × 10^9^	5.77 × 10^9^
F12	std	1.66 × 10^6^	3.04 × 10^7^	1.02 × 10^8^	3.76 × 10^7^	2.71 × 10^8^	2.21 × 10^9^	2.83 × 10^9^	3.68 × 10^9^
F12	avg	2.22 × 10^6^	2.32 × 10^7^	9.42 × 10^7^	5.82 × 10^7^	5.09 × 10^8^	7.16 × 10^9^	1.34 × 10^10^	1.27 × 10^10^
F13	min	2.67 × 10^3^	2.00 × 10^4^	7.60 × 10^4^	5.06 × 10^5^	2.22 × 10^6^	5.78 × 10^8^	2.30 × 10^9^	5.49 × 10^8^
F13	std	1.97 × 10^4^	7.41 × 10^4^	5.31 × 10^7^	2.99 × 10^5^	1.38 × 10^7^	2.54 × 10^9^	5.17 × 10^9^	4.99 × 10^9^
F13	avg	1.94 × 10^4^	9.97 × 10^4^	1.75 × 10^7^	8.82 × 10^5^	1.28 × 10^7^	3.62 × 10^9^	1.04 × 10^10^	9.22 × 10^9^
F14	min	1.07 × 10^4^	2.92 × 10^3^	8.09 × 10^3^	9.80 × 10^4^	6.07 × 10^4^	6.17 × 10^4^	4.60 × 10^4^	1.18 × 10^5^
F14	std	1.08 × 10^5^	2.52 × 10^5^	1.06 × 10^6^	1.61 × 10^6^	2.92 × 10^6^	6.97 × 10^5^	2.17 × 10^6^	4.84 × 10^6^
F14	avg	1.24 × 10^5^	2.06 × 10^5^	7.18 × 10^5^	1.47 × 10^6^	2.64 × 10^6^	9.72 × 10^5^	2.35 × 10^6^	4.49 × 10^6^
F15	min	2.16 × 10^3^	7.01 × 10^3^	1.98 × 10^4^	4.10 × 10^4^	9.18 × 10^4^	3.77 × 10^5^	3.25 × 10^7^	1.99 × 10^7^
F15	std	7.27 × 10^3^	2.62 × 10^4^	5.95 × 10^6^	7.83 × 10^4^	5.96 × 10^6^	1.35 × 10^7^	5.93 × 10^8^	4.30 × 10^8^
F15	avg	8.28 × 10^3^	2.90 × 10^4^	2.22 × 10^6^	1.32 × 10^5^	4.52 × 10^6^	8.78 × 10^6^	6.86 × 10^8^	5.00 × 10^8^
F16	min	2.29 × 10^3^	2.89 × 10^3^	2.11 × 10^3^	3.15 × 10^3^	2.96 × 10^3^	3.65 × 10^3^	4.17 × 10^3^	3.39 × 10^3^
F16	std	3.67 × 10^2^	5.77 × 10^2^	4.24 × 10^2^	3.33 × 10^2^	5.73 × 10^2^	2.63 × 10^2^	1.01 × 10^3^	1.31 × 10^3^
F16	avg	2.89 × 10^3^	3.86 × 10^3^	2.64 × 10^3^	3.66 × 10^3^	4.16 × 10^3^	4.04 × 10^3^	5.62 × 10^3^	6.12 × 10^3^
F17	min	1.92 × 10^3^	2.22 × 10^3^	1.86 × 10^3^	2.24 × 10^3^	2.18 × 10^3^	2.39 × 10^3^	2.45 × 10^3^	2.93 × 10^3^
F17	std	2.99 × 10^2^	3.10 × 10^2^	1.63 × 10^2^	2.77 × 10^2^	3.23 × 10^2^	2.55 × 10^2^	3.08 × 10^3^	4.11 × 10^3^
F17	avg	2.59 × 10^3^	2.89 × 10^3^	2.11 × 10^3^	2.69 × 10^3^	2.76 × 10^3^	2.91 × 10^3^	5.30 × 10^3^	5.90 × 10^3^
F18	min	6.90 × 10^4^	8.03 × 10^4^	8.54 × 10^4^	5.26 × 10^4^	4.48 × 10^5^	7.97 × 10^5^	9.17 × 10^5^	1.33 × 10^6^
F18	std	1.55 × 10^6^	1.59 × 10^6^	2.41 × 10^6^	4.62 × 10^6^	1.63 × 10^7^	4.46 × 10^6^	4.61 × 10^7^	1.05 × 10^8^
F18	avg	1.00 × 10^6^	1.35 × 10^6^	2.41 × 10^6^	4.62 × 10^6^	1.52 × 10^7^	6.73 × 10^6^	4.11 × 10^7^	7.81 × 10^7^
F19	min	2.19 × 10^3^	3.27 × 10^5^	1.50 × 10^4^	1.11 × 10^5^	5.02 × 10^5^	2.03 × 10^7^	5.28 × 10^7^	6.09 × 10^7^
F19	std	4.56 × 10^3^	6.84 × 10^5^	1.20 × 10^6^	1.57 × 10^6^	2.35 × 10^7^	1.83 × 10^8^	4.36 × 10^8^	6.70 × 10^8^
F19	avg	5.88 × 10^3^	1.04 × 10^6^	1.02 × 10^6^	1.68 × 10^6^	1.91 × 10^7^	2.03 × 10^8^	6.19 × 10^8^	7.73 × 10^8^
F20	min	2.34 × 10^3^	2.21 × 10^3^	2.17 × 10^3^	2.43 × 10^3^	2.34 × 10^3^	2.52 × 10^3^	2.55 × 10^3^	2.55 × 10^3^
F20	std	2.33 × 10^2^	3.75 × 10^2^	2.02 × 10^2^	1.99 × 10^2^	2.22 × 10^2^	1.45 × 10^2^	2.06 × 10^2^	2.03 × 10^2^
F20	avg	2.75 × 10^3^	2.98 × 10^3^	2.48 × 10^3^	2.80 × 10^3^	2.94 × 10^3^	2.81 × 10^3^	3.04 × 10^3^	3.03 × 10^3^
F21	min	2.46 × 10^3^	2.54 × 10^3^	2.35 × 10^3^	2.46 × 10^3^	2.53 × 10^3^	2.33 × 10^3^	2.66 × 10^3^	2.62 × 10^3^
F21	std	5.07 × 10^1^	8.66 × 10^1^	2.49 × 10^1^	4.99 × 10^1^	6.20 × 10^1^	9.77 × 10^1^	5.24 × 10^1^	5.83 × 10^1^
F21	avg	2.55 × 10^3^	2.68 × 10^3^	2.41 × 10^3^	2.58 × 10^3^	2.65 × 10^3^	2.56 × 10^3^	2.75 × 10^3^	2.72 × 10^3^
F22	min	2.30 × 10^3^	5.76 × 10^3^	2.38 × 10^3^	2.52 × 10^3^	3.20 × 10^3^	4.80 × 10^3^	8.23 × 10^3^	7.98 × 10^3^
F22	std	1.71 × 10^3^	7.69 × 10^2^	2.14 × 10^3^	1.59 × 10^3^	1.82 × 10^3^	1.05 × 10^3^	5.83 × 10^2^	7.34 × 10^2^
F22	avg	6.85 × 10^3^	7.57 × 10^3^	5.46 × 10^3^	7.47 × 10^3^	7.90 × 10^3^	6.50 × 10^3^	9.73 × 10^3^	9.60 × 10^3^
F23	min	2.85 × 10^3^	3.15 × 10^3^	2.71 × 10^3^	3.04 × 10^3^	2.97 × 10^3^	3.02 × 10^3^	3.23 × 10^3^	3.30 × 10^3^
F23	std	8.58 × 10^1^	1.83 × 10^2^	6.27 × 10^1^	1.68 × 10^2^	1.09 × 10^2^	9.82 × 10^1^	1.75 × 10^2^	1.85 × 10^2^
F23	avg	2.98 × 10^3^	3.53 × 10^3^	2.79 × 10^3^	3.34 × 10^3^	3.17 × 10^3^	3.19 × 10^3^	3.61 × 10^3^	3.74 × 10^3^
F24	min	2.96 × 10^3^	3.49 × 10^3^	2.89 × 10^3^	3.25 × 10^3^	3.13 × 10^3^	3.17 × 10^3^	3.41 × 10^3^	3.62 × 10^3^
F24	std	1.04 × 10^2^	1.30 × 10^2^	6.87 × 10^1^	1.32 × 10^2^	9.80 × 10^1^	1.31 × 10^2^	1.60 × 10^2^	2.30 × 10^2^
F24	avg	3.11 × 10^3^	3.69 × 10^3^	2.98 × 10^3^	3.49 × 10^3^	3.28 × 10^3^	3.41 × 10^3^	3.78 × 10^3^	4.03 × 10^3^
F25	min	2.88 × 10^3^	2.95 × 10^3^	2.94 × 10^3^	2.91 × 10^3^	3.07 × 10^3^	3.45 × 10^3^	4.21 × 10^3^	4.22 × 10^3^
F25	std	1.66 × 10^1^	4.49 × 10^0^	9.03 × 10^1^	4.07 × 10^1^	1.03 × 10^2^	3.65 × 10^2^	3.89 × 10^2^	5.29 × 10^2^
F25	avg	2.91 × 10^3^	2.96 × 10^3^	3.03 × 10^3^	3.01 × 10^3^	3.22 × 10^3^	4.20 × 10^3^	5.16 × 10^3^	5.14 × 10^3^
F26	min	2.84 × 10^3^	7.07 × 10^3^	3.87 × 10^3^	4.29 × 10^3^	6.61 × 10^3^	6.34 × 10^3^	9.98 × 10^3^	9.15 × 10^3^
F26	std	1.61 × 10^3^	1.72 × 10^3^	4.85 × 10^2^	1.12 × 10^3^	9.75 × 10^2^	7.48 × 10^2^	8.17 × 10^2^	1.20 × 10^3^
F26	avg	6.27 × 10^3^	9.29 × 10^3^	5.00 × 10^3^	7.92 × 10^3^	8.46 × 10^3^	8.07 × 10^3^	1.15 × 10^4^	1.15 × 10^4^
F27	min	3.22 × 10^3^	3.65 × 10^3^	3.23 × 10^3^	3.30 × 10^3^	3.27 × 10^3^	3.42 × 10^3^	3.54 × 10^3^	4.09 × 10^3^
F27	std	5.00 × 10^1^	3.91 × 10^2^	2.97 × 10^1^	1.72 × 10^2^	1.84 × 10^2^	1.72 × 10^2^	4.19 × 10^2^	3.59 × 10^2^
F27	avg	3.29 × 10^3^	4.25 × 10^3^	3.27 × 10^3^	3.57 × 10^3^	3.49 × 10^3^	3.71 × 10^3^	4.51 × 10^3^	4.81 × 10^3^
F28	min	3.21 × 10^3^	3.31 × 10^3^	3.31 × 10^3^	3.32 × 10^3^	3.41 × 10^3^	4.44 × 10^3^	6.51 × 10^3^	6.04 × 10^3^
F28	std	2.22 × 10^1^	1.28 × 10^1^	1.17 × 10^2^	7.97 × 10^1^	2.12 × 10^2^	3.59 × 10^2^	7.43 × 10^2^	8.49 × 10^2^
F28	avg	3.26 × 10^3^	3.33 × 10^3^	3.47 × 10^3^	3.49 × 10^3^	3.84 × 10^3^	5.34 × 10^3^	7.60 × 10^3^	7.56 × 10^3^
F29	min	3.78 × 10^3^	4.22 × 10^3^	3.50 × 10^3^	4.21 × 10^3^	4.24 × 10^3^	4.42 × 10^3^	5.09 × 10^3^	5.21 × 10^3^
F29	std	3.50 × 10^2^	4.46 × 10^2^	2.42 × 10^2^	4.96 × 10^2^	6.35 × 10^2^	2.49 × 10^2^	1.76 × 10^3^	2.42 × 10^3^
F29	avg	4.26 × 10^3^	5.15 × 10^3^	3.97 × 10^3^	5.05 × 10^3^	5.32 × 10^3^	5.02 × 10^3^	7.88 × 10^3^	8.33 × 10^3^
F30	min	7.60 × 10^3^	7.57 × 10^4^	2.00 × 10^6^	3.28 × 10^6^	9.35 × 10^6^	4.46 × 10^7^	3.31 × 10^8^	2.07 × 10^8^
F30	std	1.44 × 10^4^	3.60 × 10^6^	9.69 × 10^6^	4.64 × 10^6^	6.15 × 10^7^	2.03 × 10^8^	1.01 × 10^9^	1.02 × 10^9^
F30	avg	2.12 × 10^4^	4.55 × 10^6^	1.13 × 10^7^	8.59 × 10^6^	7.60 × 10^7^	2.09 × 10^8^	1.62 × 10^9^	1.61 × 10^9^

**Table 3:** The test results of the 8 algorithms improved by different strategies (D = 50).

		ASFFOX	FOX	GWO	HHO	WOA	DBO	COA	OOA
F1	min	8.99 × 10^6^	2.11 × 10^8^	2.16 × 10^9^	2.77 × 10^9^	1.38 × 10^10^	6.51 × 10^10^	9.29 × 10^10^	9.42 × 10^10^
F1	std	1.13 × 10^7^	4.24 × 10^8^	4.45 × 10^9^	1.68 × 10^9^	4.07 × 10^9^	6.66 × 10^9^	8.38 × 10^9^	9.22 × 10^9^
F1	avg	2.21 × 10^7^	7.44 × 10^8^	1.22 × 10^10^	5.59 × 10^9^	2.01 × 10^10^	7.77 × 10^10^	1.12 × 10^11^	1.16 × 10^11^
F3	min	1.94 × 10^5^	2.06 × 10^5^	1.28 × 10^5^	1.40 × 10^5^	1.80 × 10^5^	1.44 × 10^5^	1.53 × 10^5^	1.43 × 10^5^
F3	std	7.74 × 10^4^	6.89 × 10^4^	3.44 × 10^4^	1.50 × 10^4^	8.47 × 10^4^	5.08 × 10^4^	2.14 × 10^4^	3.45 × 10^4^
F3	avg	3.02 × 10^5^	3.57 × 10^5^	1.73 × 10^5^	1.70 × 10^5^	2.90 × 10^5^	2.45 × 10^5^	1.96 × 10^5^	2.17 × 10^5^
F4	min	5.51 × 10^2^	7.82 × 10^2^	7.35 × 10^2^	1.24 × 10^3^	3.20 × 10^3^	1.34 × 10^4^	3.10 × 10^4^	2.49 × 10^4^
F4	std	5.04 × 10^1^	6.27 × 10^1^	3.81 × 10^2^	4.61 × 10^2^	1.07 × 10^3^	2.00 × 10^3^	5.28 × 10^3^	6.61 × 10^3^
F4	avg	6.40 × 10^2^	9.09 × 10^2^	1.34 × 10^3^	1.85 × 10^3^	4.93 × 10^3^	1.78 × 10^4^	4.07 × 10^4^	3.85 × 10^4^
F5	min	7.97 × 10^2^	8.52 × 10^2^	6.87 × 10^2^	8.69 × 10^2^	9.78 × 10^2^	1.06 × 10^3^	1.13 × 10^3^	1.12 × 10^3^
F5	std	2.91 × 10^1^	1.33 × 10^1^	3.44 × 10^1^	3.59 × 10^1^	8.43 × 10^1^	2.92 × 10^1^	2.90 × 10^1^	3.23 × 10^1^
F5	avg	8.80 × 10^2^	8.80 × 10^2^	7.62 × 10^2^	9.41 × 10^2^	1.11 × 10^3^	1.11 × 10^3^	1.20 × 10^3^	1.20 × 10^3^
F6	min	6.55 × 10^2^	6.66 × 10^2^	6.14 × 10^2^	6.68 × 10^2^	6.78 × 10^2^	6.71 × 10^2^	6.95 × 10^2^	6.92 × 10^2^
F6	std	4.77 × 10^0^	5.39 × 10^0^	6.42 × 10^0^	4.36 × 10^0^	1.22 × 10^1^	6.93 × 10^0^	3.84 × 10^0^	4.65 × 10^0^
F6	avg	6.68 × 10^2^	6.73 × 10^2^	6.26 × 10^2^	6.80 × 10^2^	6.94 × 10^2^	6.90 × 10^2^	7.02 × 10^2^	7.01 × 10^2^
F7	min	1.59 × 10^3^	1.66 × 10^3^	1.02 × 10^3^	1.63 × 10^3^	1.66 × 10^3^	1.68 × 10^3^	1.95 × 10^3^	1.92 × 10^3^
F7	std	4.36 × 10^1^	3.61 × 10^1^	8.66 × 10^1^	7.66 × 10^1^	1.20 × 10^2^	6.25 × 10^1^	4.38 × 10^1^	6.65 × 10^1^
F7	avg	1.76 × 10^3^	1.75 × 10^3^	1.15 × 10^3^	1.91 × 10^3^	1.91 × 10^3^	1.81 × 10^3^	2.06 × 10^3^	2.06 × 10^3^
F8	min	1.12 × 10^3^	1.20 × 10^3^	9.74 × 10^2^	1.17 × 10^3^	1.32 × 10^3^	1.34 × 10^3^	1.42 × 10^3^	1.40 × 10^3^
F8	std	2.77 × 10^1^	1.09 × 10^1^	7.97 × 10^1^	3.71 × 10^1^	1.05 × 10^2^	3.38 × 10^1^	3.27 × 10^1^	3.13 × 10^1^
F8	avg	1.20 × 10^3^	1.22 × 10^3^	1.10 × 10^3^	1.24 × 10^3^	1.45 × 10^3^	1.40 × 10^3^	1.49 × 10^3^	1.50 × 10^3^
F9	min	1.27 × 10^4^	1.40 × 10^4^	3.70 × 10^3^	2.33 × 10^4^	2.47 × 10^4^	2.69 × 10^4^	2.53 × 10^4^	2.76 × 10^4^
F9	std	9.97 × 10^2^	1.05 × 10^3^	4.51 × 10^3^	3.54 × 10^3^	1.36 × 10^4^	3.49 × 10^3^	4.56 × 10^3^	4.18 × 10^3^
F9	avg	1.40 × 10^4^	1.50 × 10^4^	1.04 × 10^4^	3.18 × 10^4^	4.15 × 10^4^	3.45 × 10^4^	3.66 × 10^4^	3.67 × 10^4^
F10	min	6.70 × 10^3^	7.36 × 10^3^	7.15 × 10^3^	7.99 × 10^3^	1.13 × 10^4^	1.24 × 10^4^	1.37 × 10^4^	1.40 × 10^4^
F10	std	1.12 × 10^3^	8.39 × 10^2^	2.89 × 10^3^	1.04 × 10^3^	1.05 × 10^3^	9.39 × 10^2^	5.75 × 10^2^	4.56 × 10^2^
F10	avg	8.81 × 10^3^	9.05 × 10^3^	9.54 × 10^3^	1.05 × 10^4^	1.34 × 10^4^	1.45 × 10^4^	1.50 × 10^4^	1.51 × 10^4^
F11	min	1.44 × 10^3^	1.49 × 10^3^	2.91 × 10^3^	2.02 × 10^3^	5.70 × 10^3^	1.22 × 10^4^	2.35 × 10^4^	2.15 × 10^4^
F11	std	2.03 × 10^2^	3.14 × 10^3^	2.89 × 10^3^	8.65 × 10^2^	2.63 × 10^3^	1.99 × 10^3^	1.70 × 10^3^	2.16 × 10^3^
F11	avg	1.76 × 10^3^	5.55 × 10^3^	7.57 × 10^3^	3.13 × 10^3^	9.43 × 10^3^	1.58 × 10^4^	2.74 × 10^4^	2.74 × 10^4^
F12	min	3.03 × 10^6^	2.71 × 10^7^	4.35 × 10^7^	3.56 × 10^8^	1.93 × 10^9^	2.35 × 10^10^	4.81 × 10^10^	4.62 × 10^10^
F12	std	1.10 × 10^7^	2.15 × 10^8^	1.33 × 10^9^	5.39 × 10^8^	1.85 × 10^9^	9.26 × 10^9^	1.69 × 10^10^	1.88 × 10^10^
F12	avg	1.95 × 10^7^	1.76 × 10^8^	1.59 × 10^9^	1.04 × 10^9^	4.69 × 10^9^	4.40 × 10^10^	8.44 × 10^10^	8.64 × 10^10^
F13	min	9.06 × 10^3^	2.68 × 10^4^	1.87 × 10^6^	5.38 × 10^6^	1.10 × 10^8^	7.98 × 10^9^	2.11 × 10^10^	2.12 × 10^10^
F13	std	2.27 × 10^4^	1.11 × 10^5^	1.18 × 10^9^	4.82 × 10^7^	3.23 × 10^8^	5.86 × 10^9^	1.96 × 10^10^	1.66 × 10^10^
F13	avg	3.75 × 10^4^	1.51 × 10^5^	5.52 × 10^8^	3.84 × 10^7^	4.96 × 10^8^	1.77 × 10^10^	5.10 × 10^10^	5.64 × 10^10^
F14	min	1.55 × 10^5^	7.53 × 10^4^	1.41 × 10^4^	1.36 × 10^6^	7.92 × 10^5^	1.34 × 10^6^	1.40 × 10^7^	1.22 × 10^7^
F14	std	7.26 × 10^5^	4.31 × 10^5^	2.81 × 10^6^	5.64 × 10^6^	8.49 × 10^6^	9.84 × 10^6^	7.09 × 10^7^	1.16 × 10^8^
F14	avg	1.03 × 10^6^	4.93 × 10^5^	2.12 × 10^6^	6.34 × 10^6^	9.99 × 10^6^	9.93 × 10^6^	9.94 × 10^7^	1.64 × 10^8^
F15	min	3.54 × 10^3^	1.23 × 10^4^	5.48 × 10^4^	2.22 × 10^5^	2.95 × 10^6^	4.90 × 10^8^	4.22 × 10^9^	2.49 × 10^9^
F15	std	9.14 × 10^3^	3.69 × 10^4^	2.08 × 10^8^	5.03 × 10^5^	6.33 × 10^7^	1.13 × 10^9^	3.55 × 10^9^	3.74 × 10^9^
F15	avg	1.88 × 10^4^	3.16 × 10^4^	6.39 × 10^7^	1.36 × 10^6^	6.03 × 10^7^	2.33 × 10^9^	9.48 × 10^9^	9.61 × 10^9^
F16	min	2.84 × 10^3^	4.25 × 10^3^	2.53 × 10^3^	3.18 × 10^3^	4.73 × 10^3^	5.24 × 10^3^	6.08 × 10^3^	6.70 × 10^3^
F16	std	5.57 × 10^2^	6.40 × 10^2^	5.99 × 10^2^	6.16 × 10^2^	8.83 × 10^2^	5.23 × 10^2^	1.84 × 10^3^	1.58 × 10^3^
F16	avg	4.01 × 10^3^	5.00 × 10^3^	3.40 × 10^3^	4.70 × 10^3^	6.52 × 10^3^	6.00 × 10^3^	1.01 × 10^4^	9.56 × 10^3^
F17	min	2.72 × 10^3^	3.30 × 10^3^	2.41 × 10^3^	3.16 × 10^3^	3.48 × 10^3^	3.89 × 10^3^	4.61 × 10^3^	5.36 × 10^3^
F17	std	4.18 × 10^2^	2.64 × 10^2^	2.97 × 10^2^	4.15 × 10^2^	7.19 × 10^2^	5.29 × 10^2^	1.52 × 10^4^	9.80 × 10^3^
F17	avg	3.58 × 10^3^	3.73 × 10^3^	3.06 × 10^3^	3.74 × 10^3^	4.57 × 10^3^	5.23 × 10^3^	1.64 × 10^4^	1.43 × 10^4^
F18	min	3.27 × 10^5^	1.47 × 10^5^	5.76 × 10^5^	1.61 × 10^6^	6.72 × 10^6^	2.21 × 10^6^	3.13 × 10^7^	4.01 × 10^7^
F18	std	2.59 × 10^6^	2.24 × 10^6^	1.37 × 10^7^	8.33 × 10^6^	5.27 × 10^7^	2.03 × 10^7^	9.57 × 10^7^	1.43 × 10^8^
F18	avg	3.88 × 10^6^	2.32 × 10^6^	1.06 × 10^7^	1.20 × 10^7^	6.77 × 10^7^	3.06 × 10^7^	1.87 × 10^8^	2.23 × 10^8^
F19	min	5.46 × 10^3^	1.36 × 10^5^	1.50 × 10^5^	3.22 × 10^5^	1.15 × 10^6^	2.00 × 10^8^	1.48 × 10^9^	9.04 × 10^8^
F19	std	1.27 × 10^4^	1.92 × 10^6^	1.56 × 10^7^	2.02 × 10^6^	1.51 × 10^7^	1.19 × 10^9^	1.60 × 10^9^	1.96 × 10^9^
F19	avg	2.32 × 10^4^	2.00 × 10^6^	1.01 × 10^7^	1.90 × 10^6^	1.56 × 10^7^	1.90 × 10^9^	3.81 × 10^9^	4.12 × 10^9^
F20	min	3.19 × 10^3^	3.47 × 10^3^	2.58 × 10^3^	2.65 × 10^3^	3.21 × 10^3^	3.19 × 10^3^	3.84 × 10^3^	3.65 × 10^3^
F20	std	2.83 × 10^2^	2.09 × 10^2^	5.13 × 10^2^	3.29 × 10^2^	3.66 × 10^2^	3.21 × 10^2^	2.02 × 10^2^	2.01 × 10^2^
F20	avg	3.70 × 10^3^	3.90 × 10^3^	3.24 × 10^3^	3.59 × 10^3^	4.01 × 10^3^	3.95 × 10^3^	4.27 × 10^3^	4.10 × 10^3^
F21	min	2.58 × 10^3^	2.90 × 10^3^	2.50 × 10^3^	2.69 × 10^3^	2.91 × 10^3^	2.90 × 10^3^	3.04 × 10^3^	3.09 × 10^3^
F21	std	1.19 × 10^2^	8.60 × 10^1^	6.70 × 10^1^	8.13 × 10^1^	1.02 × 10^2^	4.02 × 10^1^	9.88 × 10^1^	9.53 × 10^1^
F21	avg	2.86 × 10^3^	3.11 × 10^3^	2.57 × 10^3^	2.94 × 10^3^	3.08 × 10^3^	2.99 × 10^3^	3.22 × 10^3^	3.23 × 10^3^
F22	min	9.14 × 10^3^	8.93 × 10^3^	7.54 × 10^3^	1.13 × 10^4^	1.26 × 10^4^	1.47 × 10^4^	1.52 × 10^4^	1.59 × 10^4^
F22	std	1.05 × 10^3^	9.96 × 10^2^	8.29 × 10^2^	9.13 × 10^2^	1.25 × 10^3^	7.82 × 10^2^	4.88 × 10^2^	4.98 × 10^2^
F22	avg	1.08 × 10^4^	1.09 × 10^4^	1.01 × 10^4^	1.25 × 10^4^	1.50 × 10^4^	1.64 × 10^4^	1.68 × 10^4^	1.69 × 10^4^
F23	min	3.28 × 10^3^	3.94 × 10^3^	2.95 × 10^3^	3.66 × 10^3^	3.55 × 10^3^	3.60 × 10^3^	4.18 × 10^3^	4.24 × 10^3^
F23	std	1.17 × 10^2^	2.22 × 10^2^	9.15 × 10^1^	2.00 × 10^2^	1.88 × 10^2^	1.32 × 10^2^	1.58 × 10^2^	1.83 × 10^2^
F23	avg	3.47 × 10^3^	4.34 × 10^3^	3.08 × 10^3^	4.06 × 10^3^	3.87 × 10^3^	3.88 × 10^3^	4.48 × 10^3^	4.59 × 10^3^
F24	min	3.41 × 10^3^	4.10 × 10^3^	3.12 × 10^3^	3.62 × 10^3^	3.69 × 10^3^	3.80 × 10^3^	4.37 × 10^3^	4.81 × 10^3^
F24	std	1.48 × 10^2^	1.48 × 10^2^	7.07 × 10^1^	2.96 × 10^2^	1.36 × 10^2^	2.08 × 10^2^	2.63 × 10^2^	4.23 × 10^2^
F24	avg	3.62 × 10^3^	4.43 × 10^3^	3.20 × 10^3^	4.43 × 10^3^	3.93 × 10^3^	4.17 × 10^3^	4.83 × 10^3^	5.54 × 10^3^
F25	min	3.11 × 10^3^	3.26 × 10^3^	3.33 × 10^3^	3.48 × 10^3^	4.30 × 10^3^	9.37 × 10^3^	1.37 × 10^4^	1.39 × 10^4^
F25	std	3.82 × 10^1^	4.29 × 10^1^	3.39 × 10^2^	1.74 × 10^2^	5.49 × 10^2^	1.08 × 10^3^	1.36 × 10^3^	1.23 × 10^3^
F25	avg	3.16 × 10^3^	3.34 × 10^3^	3.84 × 10^3^	3.72 × 10^3^	5.22 × 10^3^	1.18 × 10^4^	1.60 × 10^4^	1.62 × 10^4^
F26	min	3.41 × 10^3^	1.04 × 10^4^	5.83 × 10^3^	8.85 × 10^3^	1.17 × 10^4^	1.31 × 10^4^	1.64 × 10^4^	1.57 × 10^4^
F26	std	2.48 × 10^3^	6.50 × 10^2^	7.84 × 10^2^	1.07 × 10^3^	1.24 × 10^3^	7.33 × 10^2^	6.03 × 10^2^	8.07 × 10^2^
F26	avg	9.50 × 10^3^	1.22 × 10^4^	7.04 × 10^3^	1.17 × 10^4^	1.48 × 10^4^	1.45 × 10^4^	1.77 × 10^4^	1.74 × 10^4^
F27	min	3.48 × 10^3^	4.85 × 10^3^	3.43 × 10^3^	4.27 × 10^3^	3.89 × 10^3^	4.09 × 10^3^	5.70 × 10^3^	6.76 × 10^3^
F27	std	1.61 × 10^2^	8.89 × 10^2^	1.36 × 10^2^	6.09 × 10^2^	6.68 × 10^2^	3.63 × 10^2^	5.76 × 10^2^	6.55 × 10^2^
F27	avg	3.77 × 10^3^	6.06 × 10^3^	3.72 × 10^3^	5.04 × 10^3^	5.04 × 10^3^	5.12 × 10^3^	6.76 × 10^3^	8.03 × 10^3^
F28	min	3.40 × 10^3^	4.02 × 10^3^	3.78 × 10^3^	3.96 × 10^3^	5.21 × 10^3^	7.25 × 10^3^	1.08 × 10^4^	1.14 × 10^4^
F28	std	6.97 × 10^1^	2.05 × 10^2^	4.68 × 10^2^	3.85 × 10^2^	4.78 × 10^2^	8.42 × 10^2^	1.43 × 10^3^	1.50 × 10^3^
F28	avg	3.50 × 10^3^	4.24 × 10^3^	4.68 × 10^3^	4.80 × 10^3^	6.00 × 10^3^	8.91 × 10^3^	1.34 × 10^4^	1.39 × 10^4^
F29	min	4.33 × 10^3^	5.84 × 10^3^	4.37 × 10^3^	5.98 × 10^3^	7.15 × 10^3^	7.42 × 10^3^	1.89 × 10^4^	1.43 × 10^4^
F29	std	4.46 × 10^2^	1.17 × 10^3^	5.03 × 10^2^	9.12 × 10^2^	1.86 × 10^3^	3.55 × 10^3^	9.79 × 10^4^	2.14 × 10^5^
F29	avg	5.23 × 10^3^	7.26 × 10^3^	5.01 × 10^3^	7.04 × 10^3^	9.65 × 10^3^	1.28 × 10^4^	1.14 × 10^5^	1.49 × 10^5^
F30	min	1.01 × 10^6^	4.57 × 10^7^	8.46 × 10^7^	5.05 × 10^7^	1.06 × 10^8^	1.24 × 10^9^	2.62 × 10^9^	4.13 × 10^9^
F30	std	9.77 × 10^5^	4.12 × 10^7^	9.15 × 10^7^	8.69 × 10^7^	1.43 × 10^8^	1.15 × 10^9^	3.12 × 10^9^	2.68 × 10^9^
F30	avg	1.82 × 10^6^	9.67 × 10^7^	1.92 × 10^8^	1.42 × 10^8^	3.27 × 10^8^	2.91 × 10^9^	9.61 × 10^9^	9.12 × 10^9^

**Table 4:** Rank sum test (D = 30).

	FOX	GWO	HHO	WOA	DBO	COA	OOA
F1	5.00 × 10^−9^	3.02 × 10^−11^	3.02 × 10^−11^	3.02 × 10^−11^	3.02 × 10^−11^	3.02 × 10^−11^	3.02 × 10^−11^
F3	2.81 × 10^−2^	1.84 × 10^−2^	5.01 × 10^−1^	3.02 × 10^−11^	1.46 × 10^−10^	3.02 × 10^−11^	3.02 × 10^−11^
F4	1.10 × 10^−8^	1.46 × 10^−10^	1.78 × 10^−10^	3.02 × 10^−11^	3.02 × 10^−11^	3.02 × 10^−11^	3.02 × 10^−11^
F5	9.35 × 10^−1^	7.39 × 10^−11^	2.38 × 10^−3^	6.36 × 10^−5^	1.20 × 10^−8^	3.02 × 10^−11^	6.07 × 10^−11^
F6	5.07 × 10^−10^	3.02 × 10^−11^	2.38 × 10^−7^	8.10 × 10^−10^	5.49 × 10^−11^	3.02 × 10^−11^	3.02 × 10^−11^
F7	1.78 × 10^−4^	3.02 × 10^−11^	3.50 × 10^−3^	5.83 × 10^−3^	4.68 × 10^−2^	1.55 × 10^−9^	3.34 × 10^−11^
F8	2.42 × 10^−2^	3.34 × 10^−11^	7.62 × 10^−1^	1.09 × 10^−10^	4.50 × 10^−11^	3.02 × 10^−11^	3.02 × 10^−11^
F9	1.86 × 10^−6^	6.52 × 10^−9^	3.02 × 10^−11^	3.02 × 10^−11^	3.34 × 10^−11^	3.02 × 10^−11^	3.02 × 10^−11^
F10	4.46 × 10^−1^	7.29 × 10^−3^	3.51 × 10^−2^	8.99 × 10^−11^	3.34 × 10^−11^	3.02 × 10^−11^	3.34 × 10^−11^
F11	4.20 × 10^−10^	3.02 × 10^−11^	3.34 × 10^−11^	3.02 × 10^−11^	3.02 × 10^−11^	3.02 × 10^−11^	3.02 × 10^−11^
F12	1.29 × 10^−9^	3.34 × 10^−11^	3.34 × 10^−11^	3.02 × 10^−11^	3.02 × 10^−11^	3.02 × 10^−11^	3.02 × 10^−11^
F13	4.69 × 10^−8^	3.69 × 10^−11^	3.02 × 10^−11^	3.02 × 10^−11^	3.02 × 10^−11^	3.02 × 10^−11^	3.02 × 10^−11^
F14	6.95 × 10^−1^	5.75 × 10^−2^	5.97 × 10^−9^	8.48 × 10^−9^	2.39 × 10^−8^	5.07 × 10^−10^	1.09 × 10^−10^
F15	3.81 × 10^−7^	6.07 × 10^−11^	3.02 × 10^−11^	3.02 × 10^−11^	3.02 × 10^−11^	3.02 × 10^−11^	3.02 × 10^−11^
F16	7.77 × 10^−9^	1.70 × 10^−2^	2.03 × 10^−9^	6.12 × 10^−10^	3.02 × 10^−11^	3.02 × 10^−11^	3.34 × 10^−11^
F17	1.44 × 10^−3^	2.60 × 10^−8^	3.11 × 10^−1^	9.33 × 10^−2^	2.39 × 10^−4^	2.03 × 10^−9^	8.99 × 10^−11^
F18	2.28 × 10^−1^	1.41 × 10^−4^	2.00 × 10^−5^	4.57 × 10^−9^	4.57 × 10^−9^	2.37 × 10^−10^	9.92 × 10^−11^
F19	3.02 × 10^−11^	3.69 × 10^−11^	3.02 × 10^−11^	3.02 × 10^−11^	3.02 × 10^−11^	3.02 × 10^−11^	3.02 × 10^−11^
F20	1.17 × 10^−2^	4.64 × 10^−5^	3.48 × 10^−1^	3.03 × 10^−3^	2.23 × 10^−1^	2.00 × 10^−5^	4.08 × 10^−5^
F21	5.53 × 10^−8^	3.69 × 10^−11^	5.55 × 10^−2^	1.73 × 10^−7^	1.45 × 10^−1^	3.02 × 10^−11^	6.07 × 10^−11^
F22	1.33 × 10^−1^	3.56 × 10^−4^	2.15 × 10^−2^	3.34 × 10^−3^	5.32 × 10^−3^	4.50 × 10^−11^	1.78 × 10^−10^
F23	3.69 × 10^−11^	1.29 × 10^−9^	2.37 × 10^−10^	1.85 × 10^−8^	2.23 × 10^−9^	3.02 × 10^−11^	3.02 × 10^−11^
F24	3.02 × 10^−11^	1.03 × 10^−6^	6.07 × 10^−11^	3.52 × 10^−7^	8.89 × 10^−10^	3.02 × 10^−11^	3.02 × 10^−11^
F25	3.02 × 10^−11^	3.69 × 10^−11^	1.33 × 10^−10^	3.02 × 10^−11^	3.02 × 10^−11^	3.02 × 10^−11^	3.02 × 10^−11^
F26	1.31 × 10^−8^	2.43 × 10^−5^	8.29 × 10^−6^	1.16 × 10^−7^	8.20 × 10^−7^	3.02 × 10^−11^	3.02 × 10^−11^
F27	3.02 × 10^−11^	3.92 × 10^−2^	3.82 × 10^−10^	7.69 × 10^−8^	3.34 × 10^−11^	3.02 × 10^−11^	3.02 × 10^−11^
F28	3.02 × 10^−11^	3.02 × 10^−11^	3.02 × 10^−11^	3.02 × 10^−11^	3.02 × 10^−11^	3.02 × 10^−11^	3.02 × 10^−11^
F29	2.67 × 10^−9^	1.52 × 10^−3^	1.25 × 10^−7^	1.69 × 10^−9^	1.07 × 10^−9^	3.02 × 10^−11^	3.02 × 10^−11^
F30	3.02 × 10^−11^	3.02 × 10^−11^	3.02 × 10^−11^	3.02 × 10^−11^	3.02 × 10^−11^	3.02 × 10^−11^	3.02 × 10^−11^

**Table 5:** Design issues with I-beams solving results.

Algorithms	*X*_1_	*X*_2_	*X*_3_	*X*_4_	Best	Std	Mean
ASFFOX	5.0000 × 10^1^	8.0000 × 10^1^	1.7647 × 10^0^	5.0000 × 10^0^	−6.6260 × 10^−3^	8.8989 × 10^−19^	−6.6260 × 10^−3^
FOX	3.7339 × 10^1^	8.0000 × 10^1^	2.0730 × 10^0^	5.0000 × 10^0^	−6.6260 × 10^−3^	7.1772 × 10^−4^	−6.6260 × 10^−3^
GWO	5.0000 × 10^1^	8.0000 × 10^1^	1.7644 × 10^0^	5.0000 × 10^0^	−6.6260 × 10^−3^	3.0976 × 10^−8^	−6.6260 × 10^−3^
HHO	5.0000 × 10^1^	8.0000 × 10^1^	1.7647 × 10^0^	5.0000 × 10^0^	−6.6260 × 10^−3^	1.4070 × 10^−18^	−6.6260 × 10^−3^
WOA	5.0000 × 10^1^	8.0000 × 10^1^	1.7647 × 10^0^	5.0000 × 10^0^	−6.6260 × 10^−3^	4.0068 × 10^−11^	−6.6260 × 10^−3^
DBO	5.0000 × 10^1^	8.0000 × 10^1^	9.0000 × 10^−1^	5.0000 × 10^0^	−6.6260 × 10^−3^	5.0166 × 10^−5^	−6.6260 × 10^−3^
COA	5.0000 × 10^1^	8.0000 × 10^1^	1.7647 × 10^0^	5.0000 × 10^0^	−6.6260 × 10^−3^	4.2275 × 10^−10^	−6.6260 × 10^−3^
OOA	1.9519 × 10^1^	7.8671 × 10^1^	2.6929 × 10^0^	3.1535 × 10^0^	−7.9739 × 10^−3^	3.4607 × 10^−3^	−1.1372 × 10^−2^

**Table 6:** Speed reducer optimization design problem for eight algorithms.

	*X*_1_	*X*_2_	*X*_3_	*X*_4_	*X*_5_	*X*_6_	*X*_7_	Best	Std	Mean
ASFFOX	3.5000 × 10^0^	7.0000 × 10^−1^	1.7000 × 10^1^	7.3000 × 10^0^	7.7153 × 10^0^	3.3505 × 10^0^	5.2867 × 10^0^	2.9944 × 10^3^	3.0942 × 10^−5^	2.9944 × 10^3^
FOX	3.5014 × 10^0^	7.0000 × 10^−1^	1.7000 × 10^1^	7.6589 × 10^0^	7.7221 × 10^0^	3.3525 × 10^0^	5.2875 × 10^0^	2.9993 × 10^3^	5.7334 × 10^1^	3.0190 × 10^3^
GWO	3.5003 × 10^0^	7.0000 × 10^−1^	1.7000 × 10^1^	7.7211 × 10^0^	7.8097 × 10^0^	3.3524 × 10^0^	5.2877 × 10^0^	3.0015 × 10^3^	4.3596 × 10^0^	3.0096 × 10^3^
HHO	3.5377 × 10^0^	7.0000 × 10^−1^	1.7000 × 10^1^	7.3583 × 10^0^	7.7607 × 10^0^	3.3507 × 10^0^	5.2897 × 10^0^	3.0127 × 10^3^	9.1195 × 10^1^	3.0756 × 10^3^
WOA	3.5000 × 10^0^	7.0000 × 10^−1^	1.7000 × 10^1^	7.3000 × 10^0^	7.8222 × 10^0^	3.3537 × 10^0^	5.2921 × 10^0^	3.0010 × 10^3^	6.4486 × 10^2^	3.4228 × 10^3^
DBO	3.5043 × 10^0^	7.0000 × 10^−1^	1.7000 × 10^1^	7.3000 × 10^0^	7.7939 × 10^0^	3.4803 × 10^0^	5.2912 × 10^0^	3.0351 × 10^3^	5.3093 × 10^2^	3.2912 × 10^3^
COA	3.5000 × 10^0^	7.0000 × 10^−1^	1.7254 × 10^1^	7.3000 × 10^0^	8.0382 × 10^0^	3.3786 × 10^0^	5.3214 × 10^0^	3.0753 × 10^3^	3.2240 × 10^13^	1.7304 × 10^13^
OOA	3.5306 × 10^0^	7.0159 × 10^−1^	2.3028 × 10^1^	7.4141 × 10^0^	7.8349 × 10^0^	3.4933 × 10^0^	5.3951 × 10^0^	4.3462 × 10^3^	8.6514 × 10^13^	1.4291 × 10^14^

## Data Availability

The data that support the findings of this study are available from the corresponding author upon request. There are no restrictions on data availability.
